# Longitudinal patterns of antidepressant and benzodiazepine use associated with injurious falls in older adults with depression: a retrospective cohort study

**DOI:** 10.1186/s12916-025-04325-2

**Published:** 2025-08-20

**Authors:** Grace Hsin-Min Wang, Amie J. Goodin, Rachel C. Reise, Ronald I. Shorr, Taewoo Park, Wei-Hsuan Lo-Ciganic

**Affiliations:** 1 Department of Pharmaceutical Outcomes and Policy, College of Pharmacy, University of Florida, Gainesville, FL USA; 2 College of Public Health and Health Professions and College of Medicine, University of Florida, Gainesville, FL USA; 3 North Florida/South Georgia Veterans Health System Geriatric Research Education and Clinical Center, Gainesville, FL USA; 4 Department of Psychiatry, University of Pittsburgh School of Medicine, Pittsburgh, PA USA; 5 Division of General Internal Medicine, Department of Medicine, School of Medicine, University of Pittsburgh, Center for Research On Health Care, 3609 Forbes Avenue, 2nd Floor, Pittsburgh, PA 15213 USA; 6 Center for Pharmaceutical Policy and Prescribing, University of Pittsburgh, Pittsburgh, PA USA

**Keywords:** Elderly, Depression, Falls, Fractures, Antidepressants, Benzodiazepines, Trajectory

## Abstract

**Background:**

Cross-sectional studies have shown that antidepressants (ADs) and benzodiazepines (BZDs) are commonly co-prescribed for depression, potentially increasing the risk of falls and related injuries (FRI) compared to monotherapies. However, little is known about the longitudinal dosing patterns (i.e., trajectory) of ADs and BZDs and their associated FRI risk.

**Methods:**

This retrospective cohort study used group-based multi-trajectory models to identify AD-BZD trajectories among older Medicare fee-for-service beneficiaries with depression initiating ADs with/without BZDs. We measured the standardized daily doses of AD and BZD within 84 days after AD initiation and categorized them into negligible, very-low, low, moderate, high, or very-high levels with a discontinuing, declining, increasing, or stable trend. Then, we assessed the subsequent 12-month FRI risk associated with each trajectory.

**Results:**

Among 102,750 eligible beneficiaries, the mean age was 75.5 years (SD = 7.5); 67.0% were female, 81.2% were White, and 4.9% experienced an FRI. We identified 12 distinct AD/BZD trajectories, of which 79,424 patients received AD monotherapy, and 23,326 patients received both ADs and BZDs. Compared with Group A (low discontinuing AD; 17.3% of the cohort; FRI crude incidence rate = 99.7/1000 person-year), trajectories with a higher dose or a longer duration of AD use were associated with an increased FRI risk, regardless of BZD use. The hazard ratios (HR) and 95% confidence intervals (CI) for Groups B (low declining AD; 31.0% of the cohort), C (moderate increasing AD; 23.5%), and D (high increasing AD; 5.4%) were 1.11 (1.04–1.19), 1.24 (1.16–1.32), and 1.29 (1.16–1.42), respectively. Combining ADs and BZDs at very-low doses or with declining trends did not significantly alter FRI risk compared to AD monotherapy. However, FRI risk increased when BZDs were used at low doses (either with stable or increasing trends). The HR and 95%CI for Groups J (moderate increasing AD/low stable BZD, 1.3%) and L (very-high increasing AD/low-dose increasing BZD) were 1.71 (1.41, 2.08) and 1.96 (1.53, 2.49), respectively.

**Conclusions:**

We observed a dose–response relationship between AD use and FRI risk, independent of BZD use, highlighting the importance of initiating ADs at the lowest effective dose and closely monitoring to prevent FRI.

**Supplementary Information:**

The online version contains supplementary material available at 10.1186/s12916-025-04325-2.

## Background

Depression affects approximately one-third older adults globally [1], with 75% experiencing a co-occurring anxiety disorder [2]. Antidepressants (AD) are the first-line pharmacotherapy for depression, with some guidelines recommending short-term benzodiazepine (BZD) use for managing anxiety or insomnia symptoms, especially during the first 1–4 weeks after AD initiation [3–5]. Concurrent AD and BZD use in older adults is common in clinical practice. For example, a study using US commercial insurance claims data found that the co-initiation rate of ADs and BZDs was 12.5% [5]. By contrast, long-term concomitant use of BZD is generally not recommended due to the risk of dependency and increased likelihood of adverse outcomes, including falls and related injuries (FRI) [6].

Both ADs and BZDs possess sedative side effects that can impair or exacerbate psychomotor and cognitive functions in older adults, potentially increasing the FRI risk [7, 8]. A meta-analysis indicated that the use of ADs or BZDs alone was associated with an increased FRI risk compared to no use (AD: odds ratio [OR] = 1.57, 95% confidence interval [CI] = 1.43–1.74; BZD: OR = 1.42, 95% CI = 1.04–1.56) [9]. The 2019 American Geriatric Society Beers Criteria also recommended against combining ADs and BZDs in older adults with a history of falls or fractures, suggesting that concomitant use may further elevate FRI risk [10].

Dose titration and tapering of ADs and BZDs are common in clinical practice; however, existing studies on the concurrent use of ADs and BZDs have not explored how their doses change over time. Additionally, both the dose and duration of drug use may affect the FRI risk [11, 12]. Understanding the longitudinal utilization patterns (i.e., trajectories) of ADs with or without concurrent BZD use (hereafter “AD ± BZD”) and examining their associations with FRI risk can provide valuable insights to inform safer prescribing and guide future safety monitoring programs. Therefore, we aimed to (1) identify distinct AD ± BZD longitudinal dosing patterns and covariates associated with specific trajectories, and (2) examine the association between these trajectories and the FRI risk among older adults with depression.

## Methods

This study followed the Strengthening the Reporting of Observational Studies in Epidemiology (STROBE) guideline for reporting observational research (Additional files: Table S1) [13].

### Data Sources

We used Medicare Parts A, B, and D fee-for-service claims data comprised of a 5% sample of beneficiaries (2013–2015) and a 15% modified sample with oversampling of Floridians (2016–2019). The 15% modified sample including the original 5% sample plus additional beneficiaries, which is an oversample of those residing in Florida. All beneficiaries who met the inclusion criteria were followed longitudinally from their entry into the sample until censoring. Thus, individuals from the original 5% sample continued to be followed during the 15% sample period, and the additional beneficiaries included in 2016–2019 were followed starting in 2016. Medicare covers ~ 97% of the US population aged ≥ 65 years [14].

### Study design and cohort selection

This retrospective cohort study included Medicare beneficiaries initiating ADs with/without BZDs between 2014 and 2019, given that Medicare part D started covering BZDs in 2013 [15]. The index date (i.e., day 0) was the date of the first AD fill, and beneficiaries were required to have at least one depression diagnosis within 365 days prior to the index date. We documented daily AD and BZD utilization patterns within 84 days after the index date (i.e., trajectory measurement window). The 84-day window was selected based on typical duration of first-line treatment for depression during the acute phase, as defined by the Healthcare Effectiveness Data and Information Set (HEDIS) measurement (84–114 days) [16] and consistent with clinical recommendations (6–12 weeks) from UpToDate [17].

We excluded beneficiaries who: (1) were aged < 65 years, (2) did not reside in US, (3) received hospice care or resided in a skilled nursing facility within days [−365, 84] because the caring patterns in these settings were different from community dwelling, (4) switched to Medicare Advantage plan within days [−365, 84], (5) died within days [0, 84], or (6) experienced an FRI event within days [−365, 84]. We excluded beneficiaries who died or experienced an FRI within the 84-day trajectory measurement window to ensure adequate time for exposure classification. Including these individuals could lead to misclassification, as they would not have sufficient time to establish a treatment pattern. We followed patients from day 85 to the earliest of FRI occurrence, death, transition to Medicare Advantage plans, use of hospice services or skilled nursing facilities, or end of the 1-year follow-up (Additional files: Figures S1 and S2).

### Exposure

The exposure of interest was the beneficiary’s membership in a distinct trajectory, identified using the group-based multi-trajectory modeling (GBMTM). GBMTM is a data-driven approach to identify distinct longitudinal utilization patterns across multiple medication classes concurrently and to classify individuals into trajectory groups based on the maximum likelihood estimation [18]. We applied GBMTM with censored normal distributions, appropriate for repeatedly measured, continuous scales that may be censored at some point [19]. The independent variable was the number of days after the index date, and the dependent variables were the standardized daily doses (SDD) of ADs and BZDs.

To compute SDD, we determined whether each day was covered by any AD or BZD based on dispensing dates and days supplied [20, 21]. The SDD of ADs was calculated by dividing the prescribed daily dose by the defined daily dose (DDD) (Additional files: Table S2) [22]. For BZDs, daily dose of BZDs were converted into the diazepam milligram equivalents (DME) using an equivalency conversion table (Additional files: Table S2) [23–25]. For each patient, the SDDs of individual ADs and BZDs were then summed to obtain total SDDs for ADs and BZDs, respectively. For example, if a patient took fluoxetine 20 mg twice a day (2 DDD), imipramine 75 mg daily (0.75 DDD), alprazolam 0.5 mg three times daily (15 DME), and lorazepam 5 mg daily (30 DME), the resulting SDDs would be 2.75 DDD for ADs and 45 DME for BZDs. When a dispensing occurred before the end of the previous drug supply (i.e., dispensing date plus days supplied), the refill was assumed to begin on the day after the prior supply ended. Lastly, we addressed outliers by replacing values below the 1 st percentile with the 1 st percentile value and those above the 99th percentile with the 99th percentile value [26].

In each GBMTM, we used the most flexible functional form (up to the 4th order polynomial function) of the time variable to allow trajectories to emerge from the data [27]. Outputs of GBMTM included estimated probabilities of group membership for each individual, estimated trajectory curves over time, and the proportion in each trajectory group. The trajectory models were run separately for patients receiving ADs only (n = 79,424) and those receiving both ADs and BZDs (n = 23,326). For AD initiators with concurrent BZD use, we evaluated models with varying numbers of trajectories (2 to 5) for AD and BZD use, respectively, and then combined parameters from the single-trajectory models to construct the multi-trajectory model. For those without BZD use, we modelled AD use only.

The final number of trajectories was selected based on a combination of (1) Bayesian information criteria, wherein the largest value indicates the best-fitting model; (2) Nagin’s criteria for assessing model adequacy (Additional files: Table S3); (3) a minimum of 1% of the cohort assigned to each trajectory (at least 233 patients for AD + BZD trajectories and 794 patients for AD only trajectories); (4) clinical relevance of trajectory patterns [28]. We prioritized a parsimonious number of clinically meaningful trajectories that could inform practice and policy. Nagin’s criteria for a well-fitting trajectory model include an average posterior probability of group membership ≥ 0.7, odds of correct classification of ≥ 5.0, and narrow CIs for the estimated group membership probabilities among all groups [28].

To facilitate the labeling of AD and BZD dose levels, we defined AD use as: negligible (SDD < 0.1 DDD), very-low (0.1 to < 0.5 DDD), low (0.5 to < 1 DDD), moderate (1 to < 1.5 DDD), high (1.5 to < 2 DDD) and very high dose (≥ 2 DDD) [11, 29, 30]. Similarly, we defined BZD use as negligible (SDD < 1 DME), very-low (< 5 DME), low (5 to < 10 DME), moderate (10 to < 15 DME), high (15 to < 20 DME) and very-high dose (≥ 20 DME) [31–34]. We defined a “discontinuing” pattern as a dose reduction to the negligible level, “declining” or “increasing” patterns when the absolute change exceeded 0.1 DDD for ADs or 1 DME for BZDs, and “stable” when changes remained below these thresholds.

### Outcome

Our outcome of interest was time to first FRI, including falls, bone fractures, sprains, strains, dislocations, and superficial skin injuries in head, neck or trunk, upper limb, and lower limb. In the main analysis, FRI was identified using the acute care algorithm developed and validated by Min et al. (Additional files: Table S4). This method specifically captures the most valid and severe FRIs documented in hospital or emergency department settings, aiming to maximize specificity and minimize misclassification bias (sensitivity = 62.1%, positive predictive value = 88.6%) [35, 36].

### Covariates

Covariates were measured in the year prior to the index date, including socio-demographics, health services utilization, comorbidities (Additional files: Table S5), and concomitant medication use [37]. Comorbidities were selected based on previous literature and clinical input from two clinicians (R.S. and T.P.), and were considered present if an individual had ≥ 1 diagnosis code in any position in any medical claims. The Elixhauser comorbidity score was also calculated to provide a comprehensive comorbidity assessment [38]. Use of specific medication classes was defined by ≥ 1 prescription within that class. We calculated the anticholinergic burden score to reflect the cumulative extent of anticholinergic effects [39], and defined polypharmacy as concurrent use of ≥ 5 different medications [40].

### Statistical analysis

Missing data were imputed using the mode for categorical variables and the median for continuous variables. We compared baseline characteristics across the identified trajectories using chi-square tests for categorical variables and analysis of variance for continuous variables. For factors that showed significant differences across trajectories, we further assessed their associations with trajectory group membership using a multinomial logistic regression model. Adjusted ORs with 95% CI were reported.

Given that the identified trajectory groups likely differ in patient characteristics and disease complexity, we applied stabilized inverse probability of treatment weighting (IPTW) to balance the groups [41, 42]. Since our exposure was a multi-level variable (i.e., trajectory groups), propensity scores (PS) for each subject were estimated using a generalized boosted machine algorithm [43]. To meet the positivity assumption, we trimmed subjects with extreme PS (top and bottom 5th percentiles) [44] and removed confounders responsible for positivity violations from the weight construction [45]. We also assessed overlap in PS distributions using kernel density curves. Stabilized IPTW was calculated by multiplying each subject’s weight (1/PS) by the prevalence of the assigned trajectory group [46]. This approach yields a pseudo-population in which trajectory group membership is independent of measured covariates, thereby minimizing confounding in the assessment of FRI risk [47–49]. Covariate balance was evaluated using absolute standardized mean differences (ASMDs) [50]. Imbalanced covariates (ASMD > 0.1 after weighting) were included in the final outcome models to further adjust for residual confounding [51].

We summarized baseline characteristics using mean and standard deviation (SD) for continuous variables and number and percentage for categorical variables. Crude incidence rate of FRI was calculated as the total number of events per 1,000 person-years. Since death is a competing event precluding the outcome from occurring (i.e., individuals who die can no longer experience an FRI event) [51], we used the Fine-Gray subdistribution hazard model instead of the conventional Cox proportional hazards model to estimate crude and adjusted hazard ratios (HR) with 95%CI for time to first FRI across trajectory groups. We tested the proportional hazards assumption by visual assessment of the plotted Schoenfeld residuals [52]. GBMTM was performed using the TRAJ macro in SAS (http://www.andrew.cmu.edu/user/bjones). ASMDs and Fine-Gray model were performed using R version 4.2.3. All other analyses were conducted using SAS version 9.4 (SAS Inc., Cary, NC, USA).

### Subgroup and sensitivity analyses

We performed a series of subgroup and sensitivity analyses to examine the heterogeneity of effects and ensure the robustness of our findings. For subgroup analyses, we repeated the primary analysis after stratifying the cohort by their frailty and dementia status. Frailty was determined using the claims-based frailty index developed and validated by Kim et al. [53–56]. Beneficiaries were classified as frail if their frailty index exceeded the 25th percentile of the cohort distribution. Dementia was identified using a list of validated International Classification of Diseases, Ninth Revision codes (sensitivity = 0.86, positive predictive value = 0.78) [57] and Tenth Revision codes (sensitivity = 0.68, positive predictive value = 0.77) [58].

For sensitivity analyses, we first applied an alternative algorithm maximizing the inclusion of FRI events regardless of healthcare settings (sensitivity = 0.68, positive predictive value = 0.75) [36]. Second, we extended the trajectory measurement window to 182 days (~ 6 months) and began follow-up from day 183 to examine the long-term impact of trajectory membership on the FRI risk. Finally, acknowledging the potential for residual confounding inherent in observational studies, we calculated E-values to evaluate the possible influence of unmeasured confounders [59]. E-values represent the minimum strength of association that an unmeasured confounder would need to have with both the exposure and the outcome to fully explain away the association between trajectory membership and the FRI risk [59].

## Results

### Baseline characteristics

Among the 102,750 beneficiaries (mean age = 75.5 years [SD = 7.5]; 67.0% female; 81.2% White), 8.9% experienced an FRI. The most prevalent comorbidity was hyperlipidemia (72.0%), and the most frequently used concomitant medication class was lipid-modifying agents (47.4%). Most covariates were balanced across identified trajectory groups after the IPTW approach (Table 1 and Additional files: Table S6). The average days of supply was 41.0 days (SD = 24.9) for ADs and 29.0 days (SD = 17.5) for BZDs. The mean SDD was 0.8 DDD (SD = 0.6) for ADs and 2.0 DME (SD = 1.8) for BZDs (Table 2).

**Table 1:** Patient characteristics of eligible Medicare beneficiaries: Overall and by trajectory

Trajectory groups^a^	All	A	B	C	D	E	F	G	H	I	J	K	L	ASMD^‡^ after IPTW
Total	102,750 (100.0)	17,820 (17.3)	31,824 (31.0)	24,194 (23.5)	5586 (5.4)	4161 (4.0)	717 (0.7)	7004 (6.8)	1291 (1.3)	6351 (6.2)	1308 (1.3)	1771 (1.7)	723 (0.7)	
Age, mean (SD)	75.5 (7.5)	75.8 (7.5)	76.8 (8.0)	74.7 (7.1)	73.4 (6.5)	75.3 (7.2)	73.4 (6.5)	76.2 (7.8)	74.0 (6.9)	74.6 (7.0)	73.2 (6.5)	73.3 (6.4)	72.2 (6.0)	0.07
Male, %	33.0	31.6	32.4	37.0	36.6	26.7	31.7	28.7	33.3	31.8	33.2	28.9	28.6	0.11
White, %	81.2	78.0	79.2	85.0	86.6	82.2	80.8	79.2	74.5	84.3	75.2	86.7	79.1	0.08
Dual eligibility, %	26.4	26.4	29.5	22.1	21.7	22.1	32.1	29.2	40.7	24.2	38.1	24.8	37.3	0.11
Frailty, %	10.6	9.3	13.0	9.1	7.2	9.4	10.0	13.7	13.3	10.0	9.9	5.9	5.5	0.09
Comorbidities, %														
Anxiety disorder	32.5	27.0	25.6	25.0	25.6	57.8	70.9	55.5	64.3	53.6	57.6	47.4	50.9	0.13
Bipolar disorder	2.7	2.0	2.4	2.1	3.3	3.1	6.7	3.8	8.1	2.9	7.0	5.4	9.4	0.08
Dementia	13.6	10.0	18.2	13.2	10.7	6.9	4.5	14.9	12.8	12.0	12.1	8.8	6.8	0.05
Hyperlipidemia	72.0	73.1	72.9	73.6	65.0	73.6	72.8	73.3	69.2	70.4	62.0	59.1	50.9	0.07
Osteoarthritis	34.3	36.8	36.1	32.2	26.8	38.2	43.2	36.0	36.2	32.2	32.1	23.3	22.5	0.10
Osteoporosis	13.7	14.9	14.8	11.9	9.5	17.3	15.6	15.8	14.6	13.0	10.7	8.0	9.5	0.05
Vision disorder	52.0	56.2	55.3	50.5	38.0	56.0	49.1	54.5	46.0	48.5	39.2	31.6	25.0	0.15
Elixhauser Comorbidity Index, mean (SD)	5.0 (3.0)	5.1 (3.0)	5.2 (3.0)	4.8 (2.9)	4.2 (2.8)	5.2 (3.0)	5.3 (3.0)	5.3 (3.1)	5.3 (3.1)	4.8 (3.0)	4.6 (3.1)	3.8 (2.8)	3.6 (2.7)	0.07
Comedications, %														
Anticonvulsants	13.8	11.8	14.2	12.9	13.2	13.4	17.2	16.2	21.4	14.0	20.6	15.6	17.8	0.08
Antidementia agents	7.9	4.8	10.2	8.5	7.1	3.3	3.1	8.6	7.8	7.3	8.4	6.9	9.3	0.05
Antiparkinsonian agents	3.6	2.7	3.9	3.5	3.3	2.9	2.9	4.4	6.1	3.9	4.7	4.2	5.0	0.07
Antipsychotics	5.2	3.1	4.8	4.1	6.7	4.2	7.1	7.1	16.7	7.2	15.1	9.9	16.9	0.09
Anxiolytics†	0.8	0.8	0.8	0.6	0.9	0.9	0.6	0.9	1.5	0.8	1.4	1.0	1.4	0.05
Beta blockers	33.3	32.7	34.8	32.7	27.9	35.8	38.9	36.0	33.8	32.5	30.2	24.3	26.3	0.11
Hypnotics/sedatives†	4.7	4.2	4.2	4.1	4.7	6.1	7.8	6.0	7.4	6.3	8.6	7.5	8.3	0.04
Opioids	13.6	13.1	12.3	11.5	11.5	17.2	33.8	16.2	31.4	14.6	27.3	16.7	27.1	0.09
Psychostimulants	0.7	0.4	0.5	0.6	1.5	0.8	2.2	0.8	1.5	0.8	1.9	1.5	3.7	0.02
Anticholinergic burden index, mean (SD)	1.5 (1.9)	1.2 (1.7)	1.3 (1.8)	1.2 (1.7)	1.2 (1.6)	2.0 (1.8)	2.8 (2.2)	2.2 (2.1)	2.9 (2.4)	2.0 (1.9)	2.8 (2.3)	1.8 (1.8)	2.2 (1.9)	0.17
Polypharmacy, %	10.8	8.9	11.8	10.4	8.9	9.8	13.8	12.7	15.5	11.2	14.8	9.5	11.2	0.10

To facilitate the labeling of AD and BZD dose levels for each trajectory, we defined AD use as: negligible (standardized daily dose [SDD] < 0.1 defined daily dose [DDD]), very low (0.1 to < 0.5 DDD), low (0.5 to < 1 DDD), moderate (1 to < 1.5 DDD), high (1.5 to < 2 DDD) and very high dose (≥ 2 DDD). Similarly, we defined BZD use as negligible (SDD < 1 diazepam milligram equivalent [DME]), very-low (< 5 DME), low (5 to < 10 DME), moderate (10 to < 15 DME), high (15 to < 20 DME) and very-high dose (≥ 20 DME). We defined a “discontinuing” pattern as a dose reduction to the negligible level, “declining” or “increasing” patterns when the absolute change exceeded 0.1 DDD for ADs or 1 DME for BZDs, and “stable” when changes remained below these thresholds

*Abbreviations: AD* Antidepressants, *ASMD* Absolute standardized mean difference, *BZD* Benzodiazepine, *SD* Standard deviation

^a﻿^Trajectory groups: A: low discontinuing AD (17.3% of the cohort); B: low declining AD (31.0%); C: moderate increasing AD (23.5%); D: high increasing AD (5.4%); E: low discontinuing AD/very-low declining BZD (4.0%); F: low discontinuing AD/low declining BZD (0.7%); G: low declining AD/very-low declining BZD (6.8%); H: low declining AD/low declining BZD (1.3%); I: moderate increasing AD/very-low declining BZD (6.2%); J: moderate increasing AD/low stable BZD (1.3%); K: very-high increasing AD/very-low stable BZD (1.7%); L: very-high increasing AD/low-dose increasing BZD (0.7%)

^†^Benzodiazepines were not included in the medication classes of anxiolytics and hypnotics/sedatives

^‡^Median ASMD of 66 ASMDs from group comparisons (the number of 2-combinations from 12 trajectories: $c_2^{12}\;=\;66$; (e.g., group A vs B, A vs C)

**Table 2:** Patterns of antidepressant and benzodiazepine use during 12-month trajectory measurement period by trajectory group

Trajectory groups^a^	All	A	B	C	D	E	F	G	H	I	J	K	L
**Antidepressants‡**													
**Subclass, %**													
SSRI	59.4	50.1	49.8	75.3	61.4	54.8	45.9	47.6	37.2	70.9	59.6	58.1	47.5
SNRI	11.8	11.7	11.8	9.7	14.3	12.6	17.1	12.4	14.3	10.3	15.1	16.4	25.6
TCA	3.2	5.1	4.4	1.1	2.6	5.7	6.2	4.5	6.5	1.3	3.3	2.7	3.2
**Individual drugs, %**													
Sertraline	19.8	16.5	17.0	24.6	24.5	16.7	13.0	14.8	10.8	21.7	16.5	21.0	17.7
Escitalopram	18.9	16.1	12.2	27.3	18.9	19.3	15.7	12.8	10.8	27.3	21.2	19.3	12.8
Citalopram	10.7	9.5	11.9	11.9	7.3	9.8	7.3	10.8	7.4	10.7	8.4	5.8	5.6
Mirtazapine	9.2	8.6	14.1	4.6	4.1	7.9	6.9	14.1	12.8	6.9	8.3	6.1	6.9
Trazodone	8.3	10.3	12.4	2.8	4.8	11.0	9.6	14.6	18.5	5.1	5.4	6.6	7.6
Bupropion	7.2	6.6	7.3	6.2	12.8	5.6	7.3	6.5	8.4	5.3	8.0	9.8	8.8
Duloxetine	7.2	8.3	8.5	6.5	3.0	7.9	9.6	8.5	10.3	6.3	9.4	3.4	5.7
Fluoxetine	6.2	4.7	5.0	7.9	8.0	4.7	5.4	4.3	3.8	6.5	7.3	7.6	7.1
Paroxetine	3.4	3.1	3.3	3.3	2.4	3.7	3.4	4.3	3.6	3.9	4.9	3.7	3.6
Venlafaxine	3.4	2.8	3.1	3.0	4.2	3.1	4.6	3.6	3.6	3.6	4.6	5.2	7.5
Amitriptyline	1.5	2.2	2.4	0.5	0.7	2.3	2.5	2.3	2.9	0.5	1.5	1.1	1.1
Desvenlafaxine	1.1	0.5	0.1	0.2	6.9	1.5	2.1	0.2	0.2	0.3	0.9	7.5	12.1
Doxepin	0.7	1.5	0.7	0.3	1.1	1.5	2.3	1.1	1.8	0.3	0.7	0.9	0.9
Nortriptyline	0.6	1.1	1.0	0.2	0.2	1.2	1.0	1.0	1.3	0.3	0.7	0.3	0.3
Tryptophan	0.6	5.8	0.1	0.0	0.0	0.0	1.0	0.0	1.5	0.0	0.0	0.0	0.0
Vortioxetine	0.3	0.3	0.2	0.4	0.3	0.4	1.1	0.4	0.3	0.6	1.0	0.4	0.6
Imipramine	0.2	0.3	0.2	0.2	0.4	0.5	0.2	0.1	0.2	0.1	0.2	0.3	0.7
Vilazodone	0.2	1.3	0.1	0.0	0.0	2.2	5.4	0.1	0.2	0.1	0.1	0.0	0.1
Perphenazine	0.1	0.5	0.0	0.0	0.0	0.2	0.5	0.1	0.3	0.0	0.1	0.3	0.2
Fluvoxamine	0.1	0.0	0.1	0.1	0.0	0.0	0.1	0.1	0.4	0.1	0.2	0.3	0.2
**Utilization, mean (SD)**													
Days of supply	41.0 (24.9)	27.7 (7.4)	40.1 (23.9)	44.5 (27.2)	49.4 (29.2)	29.1 (5.6)	29.1 (7.6)	39.0 (22.8)	37.7 (22.0)	42.3 (25.9)	40.8 (24.4)	46.2 (28.2)	43.1 (26.3)
SDD	0.8 (0.6)	0.7 (0.5)	0.5 (0.3)	0.9 (0.3)	1.5 (0.9)	0.7 (0.5)	0.8 (0.5)	0.5 (0.3)	0.5 (0.3)	0.9 (0.4)	1.0 (0.4)	1.5 (0.9)	1.6 (1.0)
**Benzodiazepines‡**													
**Subclass, %**													
Intermediate-acting	48.0	-	-	-	-	44.7	49.0	46.7	59.7	45.2	55.9	41.5	47.9
Short-acting	30.8	-	-	-	-	33.6	32.1	32.1	24.8	32.7	24.3	32.2	27.5
Long-acting	21.2	-	-	-	-	21.7	18.9	21.3	15.5	22.1	19.8	26.2	24.5
**Individual drugs, %**													
Lorazepam	40.9	-	-	-	-	39.6	41.1	40.4	48.8	39.6	44.7	36.1	37.1
Alprazolam	30.6	-	-	-	-	33.4	31.9	31.8	24.5	32.5	24.2	31.9	27.5
Clonazepam	14.3	-	-	-	-	11.3	14.9	12.9	13.6	13.9	16.9	17.7	22.3
Temazepam	7.1	-	-	-	-	5.1	7.9	6.2	10.9	5.6	11.1	5.5	10.8
Diazepam	4.9	-	-	-	-	8.2	3.2	5.9	1.1	5.7	1.5	6.0	1.2
Chlordiazepoxide	1.5	-	-	-	-	1.5	0.6	1.8	0.5	1.9	1.1	1.7	0.7
Clorazepate	0.5	-	-	-	-	0.7	0.0	0.6	0.4	0.6	0.4	0.7	0.3
Triazolam	0.1	-	-	-	-	0.1	0.2	0.2	0.1	0.1	0.0	0.3	0.0
Oxazepam	0.1	-	-	-	-	0.1	0.1	0.1	0.1	0.1	0.1	0.1	0.0
**Utilization, mean (SD)**													
Days of supply	29.0 (17.5)	-	-	-	-	27.8 (16.9)	32.8 (16.3)	27.9 (17.5)	29.5 (16.3)	28.1 (17.7)	30.6 (16.6)	30.5 (19.7)	31.8 (19.0)
SDD	2.0 (1.8)	-	-	-	-	1.4 (1.0)	3.6 (2.4)	1.4 (0.9)	3.4 (2.4)	1.4 (0.9)	3.4 (2.5)	1.6 (1.1)	3.8 (3.0)

To facilitate the labeling of AD and BZD dose levels for each trajectory, we defined AD use as: negligible (standardized daily dose [SDD] < 0.1 defined daily dose [DDD]), very low (0.1 to < 0.5 DDD), low (0.5 to < 1 DDD), moderate (1 to < 1.5 DDD), high (1.5 to < 2 DDD) and very high dose (≥ 2 DDD). Similarly, we defined BZD use as negligible (SDD < 1 diazepam milligram equivalent [DME]), very-low (< 5 DME), low (5 to < 10 DME), moderate (10 to < 15 DME), high (15 to < 20 DME) and very-high dose (≥ 20 DME). We defined a “discontinuing” pattern as a dose reduction to the negligible level, “declining” or “increasing” patterns when the absolute change exceeded 0.1 DDD for ADs or 1 DME for BZDs, and “stable” when changes remained below these thresholds

*Abbreviations:* *SSRI* Selective serotonin reuptake inhibitors, *SNRI* Serotonin and norepinephrine reuptake inhibitors, *TCA* Tricyclic antidepressants, *SD* Standard deviation, *SDD* Standardized daily dose

^a^Trajectory groups: A: low discontinuing AD (17.3% of the cohort); B: low declining AD (31.0%); C: moderate increasing AD (23.5%); D: high increasing AD (5.4%); E: low discontinuing AD/very-low declining BZD (4.0%); F: low discontinuing AD/low declining BZD (0.7%); G: low declining AD/very-low declining BZD (6.8%); H: low declining AD/low declining BZD (1.3%); I: moderate increasing AD/very-low declining BZD (6.2%); J: moderate increasing AD/low stable BZD (1.3%); K: very-high increasing AD/very-low stable BZD (1.7%); L: very-high increasing AD/low-dose increasing BZD (0.7%)

^‡^The medications shown in the table were the initial medications patients used (i.e., index drug)

### Covariates associated with membership in AD-BZD trajectories

**Fig. 1:**
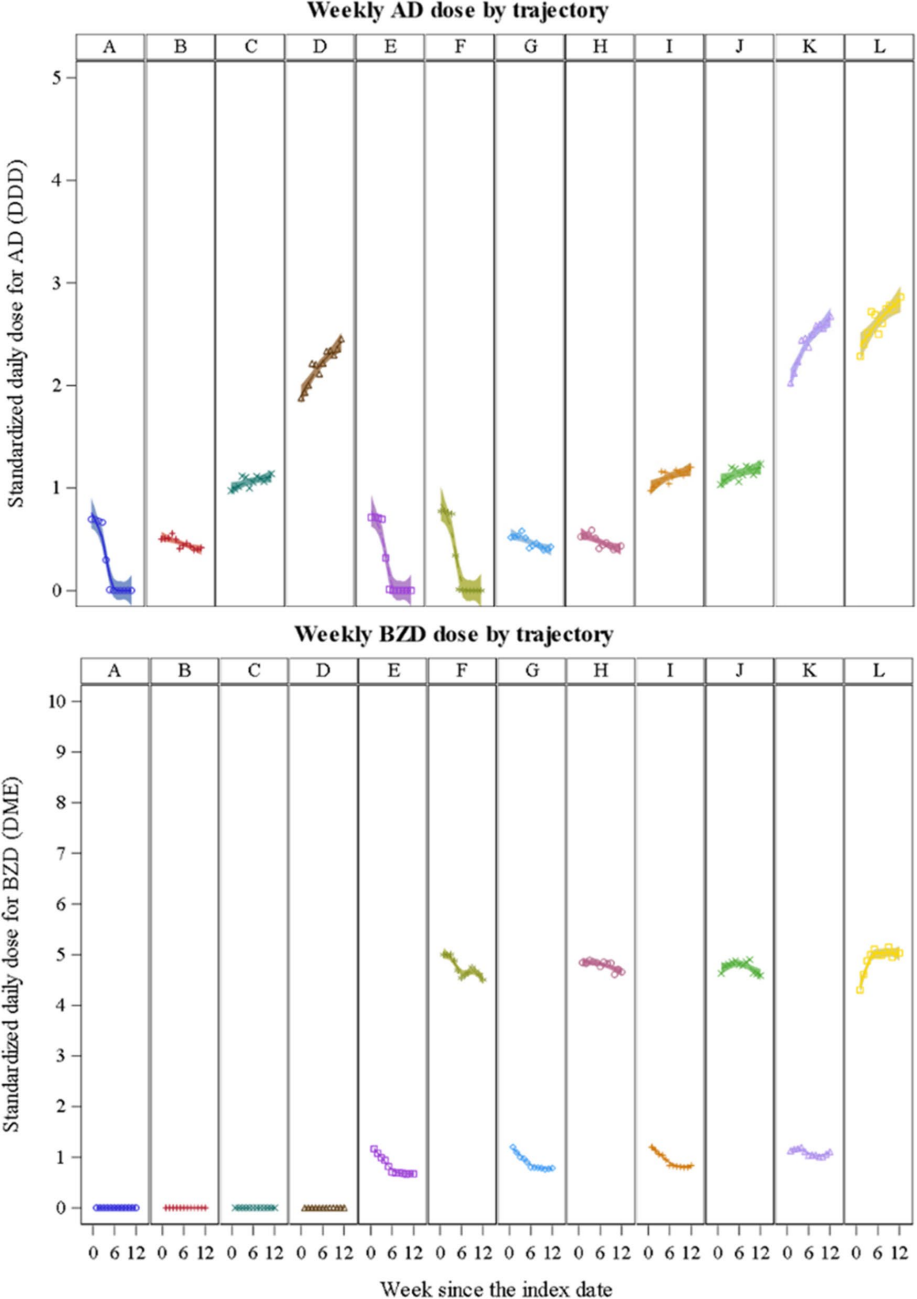
Trajectories of antidepressant and benzodiazepine utilization patterns. *Trajectory groups: A: low discontinuing AD (17.3% of the cohort); B: low declining AD (31.0%); C: moderate increasing AD (23.5%); D: high increasing AD (5.4%); E: low discontinuing AD/very-low declining BZD (4.0%); F: low discontinuing AD/low declining BZD (0.7%); G: low declining AD/very-low declining BZD (6.8%); H: low declining AD/low declining BZD (1.3%); I: moderate increasing AD/very-low declining BZD (6.2%); J: moderate increasing AD/low stable BZD (1.3%); K: very-high increasing AD/very-low stable BZD (1.7%); L: very-high increasing AD/low-dose increasing BZD (0.7%). To facilitate the labeling of AD and BZD dose levels for each trajectory, we defined AD use as: negligible (standardized daily dose [SDD] < 0.1 defined daily dose [DDD]), very low (0.1 to < 0.5 DDD), low (0.5 to < 1 DDD), moderate (1 to < 1.5 DDD), high (1.5 to < 2 DDD) and very high dose (≥ 2 DDD). Similarly, we defined BZD use as negligible (SDD < 1 diazepam milligram equivalent [DME]), very-low (< 5 DME), low (5 to < 10 DME), moderate (10 to < 15 DME), high (15 to < 20 DME) and very-high dose (≥ 20 DME). We defined a “discontinuing” pattern as a dose reduction to the negligible level, “declining” or “increasing” patterns when the absolute change exceeded 0.1 DDD for ADs or 1 DME for BZDs, and “stable” when changes remained below these thresholds

We identified 12 distinct AD-BZD trajectories (Fig. 1), organized to improve interpretability based on (1) whether BZDs were co-prescribed (AD-only: Groups A–D; AD + BZD: Groups E–L), (2) increasing AD dose levels (negligible to very-high), and (3) increasing BZD dose levels (negligible to very-high): Group A: low discontinuing AD (17.3% of the cohort); B: low declining AD (31.0%); C: moderate increasing AD (23.5%); D: high increasing AD (5.4%); E: low discontinuing AD/very-low declining BZD (4.0%); F: low discontinuing AD/low declining BZD (0.7%); G: low declining AD/very-low declining BZD (6.8%); H: low declining AD/low declining BZD (1.3%); I: moderate increasing AD/very-low declining BZD (6.2%); J: moderate increasing AD/low stable BZD (1.3%); K: very-high increasing AD/very-low stable BZD (1.7%); L: very-high increasing AD/low increasing BZD (0.7%).

Table 3 presents the covariates associated with membership in AD-BZD trajectories. Patients were more likely to be in trajectories with higher AD doses (e.g., Groups B, C, and D) if they had a diagnosis of dementia, a higher frailty index, or received medications such as anticonvulsants, antidementia agents, antiparkinsonian agents, or antipsychotics. For example, when comparing Group D vs. A, dementia was associated with an OR of 1.26 (95% CI: 1.11–1.44); frailty index, OR 1.27 (1.10–1.47); anticonvulsants, OR 1.20 (1.08–1.33); antidementia agents, OR 1.49 (1.28–1.73); antiparkinsonian agents, OR 1.30 (1.06–1.60); and antipsychotics, OR 1.69 (1.45–1.98). Conversely, patients were more likely to be in trajectories with higher BZD doses (e.g., Group F vs. A) if they had anxiety (OR = 6.22, 95%CI = 5.25–7.38) or bipolar disorders (OR = 2.28, 95% CI = 1.60–3.26), or if they received antipsychotics (OR = 1.51, 95%CI = 1.08–2.11), beta blockers (OR = 1.36, 95%CI = 1.15–1.62), or psychostimulants (OR = 3.34, 95%CI = 1.88–5.94).

**Table 3:** Covariates significantly associated with membership in AD ± BZD trajectories

Trajectory groups^a^	B	C	D	E	F	G	H	I	J	K	L
**Patient-level**											
Disability	0.85 (0.76, 0.95)	0.68 (0.60, 0.76)	0.58 (0.47, 0.71)	0.48 (0.37, 0.61)	0.37 (0.23, 0.59)	0.44 (0.36, 0.54)	0.36 (0.24, 0.52)	0.46 (0.37, 0.56)	0.38 (0.26, 0.56)	0.32 (0.21, 0.50)	0.19 (0.09, 0.39)
Metropolitan	1.14 (1.08, 1.20)	1.09 (1.03, 1.15)	1.26 (1.15, 1.38)	1.13 (1.03, 1.25)	0.99 (0.80, 1.22)	1.27 (1.17, 1.38)	1.32 (1.11, 1.57)	1.29 (1.19, 1.41)	1.56 (1.29, 1.89)	1.52 (1.29, 1.80)	1.29 (0.99, 1.66)
Frailty	1.08 (1.00, 1.17)	1.11 (1.02, 1.21)	1.27 (1.10, 1.47)	1.00 (0.86, 1.15)	1.02 (0.75, 1.38)	1.07 (0.96, 1.20)	0.96 (0.77, 1.20)	1.09 (0.96, 1.23)	1.05 (0.82, 1.34)	1.07 (0.83, 1.37)	1.18 (0.80, 1.76)
Anxiety disorder	0.99 (0.94, 1.03)	0.99 (0.95, 1.04)	1.06 (0.98, 1.13)	3.60 (3.35, 3.87)	6.22 (5.25, 7.38)	3.42 (3.22, 3.64)	4.99 (4.40, 5.66)	3.32 (3.12, 3.53)	4.24 (3.75, 4.80)	2.89 (2.61, 3.21)	3.66 (3.12, 4.30)
Bipolar disorder	1.02 (0.89, 1.18)	0.89 (0.77, 1.04)	0.91 (0.74, 1.12)	1.40 (1.11, 1.75)	2.28 (1.60, 3.26)	1.27 (1.06, 1.53)	1.62 (1.24, 2.11)	0.91 (0.74, 1.11)	1.37 (1.04, 1.80)	1.16 (0.89, 1.51)	1.54 (1.12, 2.12)
Dementia	1.57 (1.46, 1.69)	1.41 (1.30, 1.53)	1.26 (1.11, 1.44)	0.76 (0.65, 0.90)	0.51 (0.34, 0.77)	1.23 (1.10, 1.37)	1.11 (0.89, 1.39)	1.26 (1.12, 1.42)	1.30 (1.04, 1.64)	0.99 (0.79, 1.23)	0.69 (0.48, 0.99)
Hyperlipidemia	0.90 (0.86, 0.95)	0.93 (0.88, 0.98)	0.75 (0.69, 0.81)	0.91 (0.84, 1.00)	0.98 (0.81, 1.19)	0.89 (0.83, 0.96)	0.88 (0.76, 1.01)	0.79 (0.74, 0.86)	0.70 (0.61, 0.81)	0.69 (0.61, 0.78)	0.65 (0.54, 0.78)
Urinary incontinence	1.00 (0.94, 1.06)	0.95 (0.89, 1.02)	0.98 (0.88, 1.09)	0.84 (0.75, 0.95)	0.74 (0.57, 0.96)	0.87 (0.79, 0.95)	0.93 (0.77, 1.11)	0.79 (0.71, 0.87)	0.64 (0.51, 0.80)	0.86 (0.72, 1.04)	0.91 (0.68, 1.21)
Vertigo	0.89 (0.84, 0.94)	0.81 (0.76, 0.86)	0.73 (0.66, 0.81)	1.02 (0.93, 1.12)	0.87 (0.70, 1.08)	0.86 (0.80, 0.94)	0.92 (0.78, 1.09)	0.83 (0.76, 0.91)	0.71 (0.59, 0.86)	0.72 (0.60, 0.86)	0.77 (0.58, 1.03)
Antiarrhythmic agents (class 1 & 3)	0.85 (0.75, 0.97)	0.86 (0.74, 0.99)	0.65 (0.49, 0.85)	1.22 (0.98, 1.51)	1.19 (0.73, 1.94)	1.03 (0.85, 1.24)	1.19 (0.80, 1.76)	0.94 (0.77, 1.16)	0.94 (0.60, 1.49)	1.11 (0.76, 1.61)	1.89 (1.12, 3.20)
Anticonvulsants	1.16 (1.09, 1.24)	1.14 (1.06, 1.22)	1.20 (1.08, 1.33)	1.09 (0.98, 1.23)	1.01 (0.81, 1.27)	1.20 (1.10, 1.31)	1.25 (1.06, 1.48)	1.15 (1.04, 1.26)	1.29 (1.09, 1.53)	1.24 (1.06, 1.45)	1.16 (0.92, 1.45)
Antidementia agents	1.46 (1.34, 1.60)	1.63 (1.48, 1.80)	1.49 (1.28, 1.73)	0.90 (0.73, 1.11)	0.97 (0.60, 1.58)	1.45 (1.27, 1.65)	1.24 (0.96, 1.61)	1.41 (1.23, 1.63)	1.30 (1.00, 1.67)	1.58 (1.25, 2.01)	2.08 (1.50, 2.88)
Antiparkinsonian agents	1.32 (1.16, 1.50)	1.24 (1.08, 1.43)	1.30 (1.06, 1.60)	1.11 (0.88, 1.42)	0.95 (0.57, 1.58)	1.36 (1.13, 1.63)	1.65 (1.21, 2.23)	1.39 (1.15, 1.68)	1.39 (1.00, 1.92)	1.40 (1.04, 1.89)	1.58 (1.04, 2.39)
Antipsychotics	1.17 (1.05, 1.31)	1.19 (1.05, 1.34)	1.69 (1.45, 1.98)	1.23 (1.01, 1.49)	1.51 (1.08, 2.11)	1.47 (1.27, 1.70)	2.75 (2.23, 3.39)	1.75 (1.51, 2.03)	2.27 (1.83, 2.80)	1.95 (1.59, 2.40)	2.41 (1.87, 3.11)
Anxiolytics^†^	0.98 (0.79, 1.22)	0.83 (0.65, 1.05)	0.91 (0.64, 1.30)	0.37 (0.26, 0.54)	0.10 (0.04, 0.28)	0.34 (0.25, 0.46)	0.29 (0.18, 0.49)	0.29 (0.20, 0.41)	0.24 (0.14, 0.41)	0.35 (0.21, 0.60)	0.34 (0.17, 0.69)
Beta blockers	1.02 (0.97, 1.06)	1.01 (0.97, 1.06)	0.97 (0.90, 1.05)	1.14 (1.05, 1.23)	1.36 (1.15, 1.62)	1.08 (1.01, 1.15)	1.10 (0.96, 1.27)	1.02 (0.95, 1.10)	1.03 (0.89, 1.18)	0.86 (0.75, 0.97)	1.13 (0.93, 1.37)
Diuretics	1.08 (1.02, 1.13)	1.11 (1.06, 1.17)	1.09 (1.00, 1.19)	0.80 (0.73, 0.88)	0.71 (0.58, 0.88)	0.84 (0.78, 0.91)	0.79 (0.68, 0.93)	0.86 (0.80, 0.93)	0.94 (0.81, 1.10)	0.87 (0.75, 1.00)	0.67 (0.53, 0.85)
Lipid-modifying agents	1.28 (1.23, 1.34)	1.41 (1.35, 1.47)	1.42 (1.32, 1.53)	1.02 (0.95, 1.11)	0.86 (0.72, 1.02)	1.25 (1.17, 1.34)	1.08 (0.95, 1.24)	1.38 (1.29, 1.48)	1.27 (1.11, 1.45)	1.46 (1.30, 1.64)	1.26 (1.05, 1.51)
Psychostimulants	1.27 (0.95, 1.68)	1.29 (0.97, 1.73)	2.55 (1.84, 3.54)	1.80 (1.18, 2.74)	3.34 (1.88, 5.94)	1.87 (1.30, 2.69)	2.30 (1.35, 3.91)	1.49 (1.02, 2.17)	2.62 (1.62, 4.25)	1.90 (1.18, 3.04)	3.89 (2.39, 6.35)
Steroids	0.88 (0.81, 0.96)	0.85 (0.77, 0.93)	0.79 (0.67, 0.92)	1.01 (0.87, 1.17)	0.81 (0.58, 1.13)	0.93 (0.81, 1.05)	1.01 (0.79, 1.28)	0.88 (0.77, 1.01)	0.86 (0.66, 1.12)	1.01 (0.81, 1.27)	0.56 (0.36, 0.86)
Anticholinergic burden index	1.02 (1.01, 1.04)	1.03 (1.01, 1.04)	1.05 (1.02, 1.07)	1.32 (1.29, 1.34)	1.49 (1.44, 1.54)	1.34 (1.32, 1.36)	1.47 (1.43, 1.51)	1.34 (1.32, 1.36)	1.50 (1.46, 1.55)	1.35 (1.31, 1.39)	1.43 (1.37, 1.49)
**Provider-level**^§^											
Monthly patients receiving AD	0.93 (0.87, 0.99)	0.90 (0.84, 0.96)	0.97 (0.89, 1.05)	0.64 (0.57, 0.72)	0.63 (0.52, 0.77)	0.68 (0.62, 0.74)	0.67 (0.59, 0.75)	0.63 (0.58, 0.69)	0.68 (0.60, 0.76)	0.71 (0.63, 0.79)	0.66 (0.58, 0.76)
Monthly patients receiving BZD	1.08 (0.98, 1.18)	1.19 (1.08, 1.31)	1.19 (1.06, 1.34)	1.88 (1.66, 2.13)	2.06 (1.69, 2.52)	1.85 (1.67, 2.06)	1.96 (1.71, 2.25)	2.02 (1.82, 2.25)	1.81 (1.57, 2.09)	1.80 (1.56, 2.07)	1.89 (1.61, 2.22)
Monthly AD dose	0.97 (0.96, 0.98)	1.01 (1.00, 1.02)	1.04 (1.03, 1.06)	1.02 (1.01, 1.04)	1.05 (1.03, 1.07)	1.01 (1.00, 1.02)	1.04 (1.02, 1.05)	1.02 (1.01, 1.03)	1.03 (1.02, 1.04)	1.04 (1.03, 1.05)	1.04 (1.03, 1.06)

To facilitate the labeling of AD and BZD dose levels for each trajectory, we defined AD use as: negligible (standardized daily dose [SDD] < 0.1 defined daily dose [DDD]), very low (0.1 to < 0.5 DDD), low (0.5 to < 1 DDD), moderate (1 to < 1.5 DDD), high (1.5 to < 2 DDD) and very high dose (≥ 2 DDD). Similarly, we defined BZD use as negligible (SDD < 1 diazepam milligram equivalent [DME]), very-low (< 5 DME), low (5 to < 10 DME), moderate (10 to < 15 DME), high (15 to < 20 DME) and very-high dose (≥ 20 DME). We defined a “discontinuing” pattern as a dose reduction to the negligible level, “declining” or “increasing” patterns when the absolute change exceeded 0.1 DDD for ADs or 1 DME for BZDs, and “stable” when changes remained below these thresholds

Each cell represents the adjusted odds ratio for the membership in each trajectory group compared to Group A. For simplicity, we only showed patient characteristics significantly associated with the membership in each trajectory group

Abbreviations: *ACEI* Angiotensin converting enzyme inhibitor, *AD* Antidepressants, *ARB* Angiotensin receptor blocker, *BZD* Benzodiazepine, *ED* Emergency department, *NSAID* Nonsteroidal anti-inflammatory drug, *SD* Standard deviation

^a^Trajectory groups: A: low discontinuing AD (17.3% of the cohort); B: low declining AD (31.0%); C: moderate increasing AD (23.5%); D: high increasing AD (5.4%); E: low discontinuing AD/very-low declining BZD (4.0%); F: low discontinuing AD/low declining BZD (0.7%); G: low declining AD/very-low declining BZD (6.8%); H: low declining AD/low declining BZD (1.3%); I: moderate increasing AD/very-low declining BZD (6.2%); J: moderate increasing AD/low stable BZD (1.3%); K: very-high increasing AD/very-low stable BZD (1.7%); L: very-high increasing AD/low-dose increasing BZD (0.7%)

^†^Benzodiazepines were not included in the medication classes of anxiolytics and hypnotics/sedatives

^§^The provider was identified as the first physician who recorded the diagnosis of depression in the patient’s medical records. The missingness of the provider-related factors was 22.1%

### FRI risk associated with each trajectory

Compared to Group A (incidence rate = 99.7/1000 person-year), Groups B (HR = 1.11, 95%CI = 1.04, 1.19), C (HR = 1.24, 95%CI = 1.16, 1.32), and D (HR = 1.29, 95%CI = 1.16, 1.42) were more likely to experience FRI during the 1-year follow-up (Table 4). In contrast, Group E was associated with a slightly lower risk of FRI (HR = 0.84, 95%CI = 0.73, 0.96), while Group F showed no significant association (HR = 1.09, 95%CI = 0.78, 1.52). Other trajectories with higher AD and/or BZD doses than Group A were also associated with an increased FRI risk, ranging from Group G (HR = 1.15, 95% CI: 1.04–1.27) to Group L (HR = 1.96, 95% CI: 1.53–2.49). The E-values for groups with significant associations ranged from 1.47 to 3.32, suggesting moderate robustness to potential unmeasured confounding.

**Table 4:** Trajectories of antidepressant and benzodiazepine utilization patterns and 12-month risk of falls and related injuries among medicare beneficiaries (n = 102,750)

Trajectory group^a^	Number of patients (% of the cohort)	Number of FRI events	Median follow-up days (IQR)	Median days to FRI (IQR)	Incidence rate (/1000 person-year)	Crude HR (95% CI)	Adjusted HR (95% CI)	E-value
A	17,820 (17.3)	1373	365 (170)	155 (179)	99.70	Reference	Reference	Ref
B	31,824 (31.0)	2864	365 (194)	147 (181)	120.41	1.21 (1.13, 1.29)	1.11 (1.04, 1.19)	1.47
C	24,194 (23.5)	2197	365 (164)	146 (180)	116.86	1.17 (1.10, 1.25)	1.24 (1.16, 1.32)	1.78
D	5,586 (5.4)	538	365 (125)	159 (185)	118.84	1.19 (1.08, 1.32)	1.29 (1.16, 1.42)	1.90
E	4,161 (4.0)	267	365 (151)	183 (198)	81.31	0.82 (0.72, 0.93)	0.84 (0.73, 0.96)	1.21
F	717 (0.7)	63	365 (122)	133 (172)	109.32	1.10 (0.85, 1.41)	1.09 (0.78, 1.52)	1.40
G	7,004 (6.8)	615	365 (174)	162 (183)	114.81	1.15 (1.05, 1.27)	1.15 (1.04, 1.27)	1.56
H	1,291 (1.3)	143	365 (157)	168 (164)	141.33	1.42 (1.19, 1.68)	1.27 (1.02, 1.60)	1.87
I	6,351 (6.2)	612	365 (167)	129 (178)	124.47	1.25 (1.14, 1.37)	1.28 (1.16, 1.41)	1.88
J	1,308 (1.3)	167	365 (165)	149 (193)	163.91	1.64 (1.40, 1.93)	1.71 (1.41, 2.08)	2.81
K	1,771 (1.7)	210	365 (126)	146 (178)	145.68	1.46 (1.27, 1.69)	1.39 (1.18, 1.64)	2.13
L	723 (0.7)	101	365 (119)	162 (166)	170.11	1.71 (1.40, 2.09)	1.96 (1.53, 2.49)	3.32

To facilitate the labeling of AD and BZD dose levels for each trajectory, we defined AD use as: negligible (standardized daily dose [SDD] < 0.1 defined daily dose [DDD]), very low (0.1 to < 0.5 DDD), low (0.5 to < 1 DDD), moderate (1 to < 1.5 DDD), high (1.5 to < 2 DDD) and very high dose (≥ 2 DDD). Similarly, we defined BZD use as negligible (SDD < 1 diazepam milligram equivalent [DME]), very-low (< 5 DME), low (5 to < 10 DME), moderate (10 to < 15 DME), high (15 to < 20 DME) and very-high dose (≥ 20 DME). We defined a “discontinuing” pattern as a dose reduction to the negligible level, “declining” or “increasing” patterns when the absolute change exceeded 0.1 DDD for ADs or 1 DME for BZDs, and “stable” when changes remained below these thresholds

^a^Trajectory groups: A: low discontinuing AD (17.3% of the cohort); B: low declining AD (31.0%); C: moderate increasing AD (23.5%); D: high increasing AD (5.4%); E: low discontinuing AD/very-low declining BZD (4.0%); F: low discontinuing AD/low declining BZD (0.7%); G: low declining AD/very-low declining BZD (6.8%); H: low declining AD/low declining BZD (1.3%); I: moderate increasing AD/very-low declining BZD (6.2%); J: moderate increasing AD/low stable BZD (1.3%); K: very-high increasing AD/very-low stable BZD (1.7%); L: very-high increasing AD/low-dose increasing BZD (0.7%)

Subgroup analyses yielded similar results for beneficiaries without frailty and those without dementia (Additional files: Table S7). However, among beneficiaries with frailty or dementia, no significant differences in FRI risk were observed across several trajectory groups, possibly due to limited sample sizes. Findings were consistent across all sensitivity analyses (Additional files: Table S7).

## Discussion

Using large national Medicare claims data, our findings provide important insights into the initiation of ADs with/without BZDs in older adults with depression. Notably, most of our cohort was prescribed low-dose ADs with a discontinuing or declining pattern—48.3% without BZD use and 12.8% with BZD use. Among patients receiving both ADs and BZDs (22.8%), very-low declining BZD was the most common utilization pattern (17.0%). For patients initiating ADs only, higher doses and longer durations were associated with an increased FRI risk (Groups B, C, and D vs. A), regardless of BZD use. Combining ADs and BZDs at a very-low dose or with a declining trend did not significantly alter the FRI risk compared to AD monotherapy (Group F vs. A), but the risk increased when BZDs were used at low-doses with stable or increasing trends (Group J vs. C; Group L vs. D).

The majority of our study populations initiating both ADs and BZDs did not adhere to guideline recommendations against long-term BZD use [6], a persistent concern given the known risk of adverse outcomes such as falls and fractures [60]. For example, Bushnell et al. reported that 22% of patients initiating both ADs and BZDs continued receiving both medications for over six months [5]. Similarly, another study found that 14% of veterans with depression were on concurrent AD and BZD therapy for more than one year [61]. Our study adds to the existing literature by characterizing the longitudinal dosage patterns of ADs and BZDs and examining their associations with FRI risk. Notably, we observed that among older adults with depression, the most common BZD utilization pattern was very-low-dose and declining. This may reflect increasing clinical awareness of the risks associated with BZD use in this population and greater adherence to safety guidelines such as the Beers Criteria [10].

Our study demonstrated that higher-dose and longer-term of AD use were associated with an increased FRI risk. While previous research has shown that ADs may elevate FRI risk, few studies have specifically examined the impact of AD dosage. Marcum et al. found that, compared to no use, moderate-dose AD use (SDD = 1–2 DDD) was associated with an increased FRI risk in community-dwelling older adults, whereas low-dose AD use (SDD < 1 DDD) was not, suggesting a dose–response relationship between ADs and FRI risk [11]. However, that study did not focus on AD use for depression, whereas our analysis was limited to patients with a depression diagnosis to reduce confounding by indication. Similarly, Bolton et al. and Vestergaard et al. reported that fracture risk significantly increased with higher doses of selective serotonin reuptake inhibitors and tricyclic antidepressants [62, 63]. Our study extends these findings by comprehensively examining all classes of ADs and accounting for the concurrent use of multiple ADs to investigate the relationship between total AD dose and FRI risk.

Existing evidence on the association between combined AD and BZD use and FRI risks remains inconsistent, with studies varying in their definitions of concurrent use and few examining BZD dosage patterns over time. For example, a self-controlled case series study found that concurrent use of BZDs with ADs was associated with a higher risk of fractures compared to AD monotherapy (incidence rate ratio = 1.88, 95%CI = 1.66–2.12) [64]. However, the study defined concurrent BZD use as having any BZD prescription during AD treatment episodes, without accounting for BZD dose or duration [64]. In contrast, Wagner et al. reported that the policy-driven reductions in BZD use among older adults did not decrease the incidence of hip fractures, suggesting no causal relationship between BZD use and hip fractures [65]. That study defined BZD exposure as at least one BZD dispensing within one year before the policy implementation, again without considering dosage patterns. Our study adds to this body of evidence by incorporating longitudinal BZD dose trajectories. We found that combining ADs and BZDs at very-low doses or with declining trends did not significantly alter FRI risk compared to AD monotherapy. This may be due to improved management of depressive symptoms and severity, which are independently associated with increased FRI risk [66]. In such cases, we hypothesized that the therapeutic benefit of improved depression symptoms and severity may offset the anticholinergic side effects of the medications [67]. Nevertheless, our findings also showed that FRI risk increased when BZDs were used at low-doses with stable or increasing trends, underscoring the need for cautious BZD use in older adults.

Our study presents important clinical implications. First, we identified a potential dose–response relationship between AD use and FRI risk, highlighting the importance of initiating ADs at the lowest effective dose (< 1 DDD) and regularly reassessing AD doses throughout treatment. Second, though we did not observe a clear dose–response relationship between BZD use and FRI risk within the 12-month timeframe, such relationship may emerge with longer-term or higher-dose of BZD use. This finding underscores the need for ongoing surveillance and monitoring in older adults receiving both ADs and BZDs. Third, we identified key covariates associated with AD ± BZD trajectory membership, which may help clinicians pinpoint patients requiring closer monitoring. Specifically, patients with dementia and those prescribed anticonvulsants, antidementia agents, antiparkinsonian agents, or antipsychotics were more likely to receive higher AD doses for a longer duration, placing them at greater FRI risk. Therefore, extra caution is warranted when prescribing ADs in these high-risk populations.

### Limitations

First, we excluded patients who died or experienced an FRI within 84 days of AD initiation to allow adequate classification of trajectory patterns. While this could introduce selection bias—particularly if event rates are high during the trajectory measurement period—the proportion excluded was low (2.4% among patients with depression), suggesting minimal impact on the overall findings. Second, despite adjusting for a broad set of covariates, unmeasured confounding may still be present. Factors such as depression or anxiety severity, lifestyle behaviors, functional status, and living environments—none of which are captured in claims data—could influence the observed associations. Ideally, a subgroup analysis stratified by depression severity (e.g., defined by hospitalization) would help to address the bias. However, a meaningful subgroup trajectory analysis is less likely feasible due to small sample sizes in our case, especially in the concurrent AD + BZD group. Therefore, the possibility of residual confounding by indication cannot be entirely excluded. That said, our E-value analysis suggests that an unmeasured confounder would need to be strongly associated with both the exposure and the outcome to fully explain away the observed effect estimates, supporting the robustness of our findings. Third, we adjusted for time-fixed but not time-varying confounders, as the included covariates (e.g., demographics, chronic comorbidities, chronic comedication use) are generally stable and unlikely to change substantially within the 84-day trajectory measurement window. This approach reduced analytical complexity in the trajectory analysis without compromising the validity of the results. Fourth, patients with dementia are likely to develop delirium, which is also an independent risk factor of FRI. While we adjusted for baseline dementia diagnoses, delirium is an acute condition that is often underdiagnosed and poorly captured in claims data, limiting its reliability as a covariate in claims-based analyses [68]. Importantly, our study censored patients receiving care from skilled nursing facilities, who are more likely to have severe dementia and higher rates of delirium. This may help reduce confounding due to unmeasured severity of cognitive impairment. Fifth, this study focused on Medicare fee-for-service beneficiaries, which may limit the generalizability of our findings to other populations (e.g., young adults). In addition, we were unable to follow beneficiaries after they enrolled in Medicare Advantage, which covers slightly more than 50% of the Medicare population as of 2023 [69]. This may limit the generalizability of our findings to the broader Medicare population [70]. Sixth, reliance on administrative claims data may have introduced misclassification bias. For example, FRI events could be underreported if patients did not seek hospital care, and the actual medication use cannot be verified. Seventh, we were unable to account for treatment-emergent adverse effects such as hyponatremia—a known side effect of SSRIs that may increase FRI risk [71]. Because hyponatremia can develop after antidepressant initiation and is not reliably captured in claims data, its role as an intermediate factor could not be evaluated in this study. Eighth, we identified nursing facility residents using Medicare-reimbursed skilled nursing facility claims. Because Medicare does not cover long-term custodial nursing home care, our approach may not fully capture long-term care residents who receive support through Medicaid or private pay. As such, some individuals with advanced frailty or cognitive impairment may have been misclassified as community-dwelling. Ninth, baseline hypertension was not adjusted, as evidence regarding its association with increased FRI risk remains inconsistent [72]. Instead, hypotension [73] and use of antihypertensive medications [74] have been more consistently associated with elevated FRI risk. We have adjusted for these covariates in the baseline period. Tenth, while our study included dual-eligible beneficiaries, we acknowledge that Medicare claims may not fully capture certain services covered exclusively under Medicaid (e.g., long-term nursing home care, home- and community-based services), potentially leading to underestimation of some healthcare utilization. However, Medicare is the primary payer for most acute and outpatient medical care, and our exposures of interest (i.e., antidepressants and benzodiazepines) are covered under Medicare Part D and thus are expected to be well captured. Additionally, we excluded beneficiaries receiving hospice care or resided in skilled nursing facilities, as these settings may influence fall/fracture risk and confound medication exposure. Finally, dual eligibility was included as covariate and addressed through stabilized inverse probability of treatment weighting. While this remains a potential source of misclassification, the impact on our findings is likely limited given the nature of the exposures, the exclusion criteria applied, and the analytic adjustments. Despite these limitations, our sensitivity analyses yielded consistent results and E-values were large, indicating a robust association between AD ± BZD use trajectories and FRI risk.

## Conclusions

Among fee-for-service Medicare beneficiaries with depression initiating ADs, we observed a dose–response relationship between AD use and FRI risk. Combining ADs and BZDs at a very-low dose or with a declining trend did not significantly alter the FRI risk compared to AD monotherapy, but the risk increased when BZDs were used at low-doses with stable or increasing trends. Our findings highlight the importance of initiating ADs at the lowest effective dose in older adults with depression and emphasize the need for careful evaluation and monitoring to prevent FRI.

## Supplementary Information

Additional file 1: Table S1. The Strengthening the Reporting of Observational Studies in Epidemiology (STROBE) checklist. Table S2. Equivalency Conversion Table for Antidepressants and Benzodiazepines. Table S3. Nagin’s Diagnostic Criteria for Group-Based Multi-Trajectory Models of Antidepressant and Benzodiazepine Use among Medicare Beneficiaries (n=102,750). Table S4. International Classification of Diseases Clinical Modification Codes for Fall and Related Injuries. Table S5. International Classification of Diseases Clinical Modification Codes for Clinical Characteristics. Table S6. Detailed patient characteristics of eligible Medicare beneficiaries: Overall and by trajectory. Table S7. Trajectories of Antidepressant and Benzodiazepine Utilization Patterns and Risk of Falls and Related Injuries: Subgroup and Sensitivity analyses. Figure S1. Study Design Schematic Diagram. Figure S2. Cohort Selection Flowchart.

## Data Availability

The datasets generated or analyzed during this study are not publicly available due to regulations set by the CMS regarding the use of Research Identifiable Files (RIFs). Access to the data requires researchers to submit a formal data request and obtain approval through the Research Data Assistance Center (ResDAC). For more information on the request process, please visit [https://resdac.org/cms-research-identifiable-request-process-timeline].

## Abbreviations

AD: Antidepressant
ASMD: Absolute standardized mean difference
BZD: Benzodiazepine
CI: Confidence interval
DDD: Defined daily dose
DME: Diazepam milligram equivalent
FRI: Falls and related injuries
GBMTM: Group-based multi-trajectory modeling
HR: Hazard ratio
IPTW: Inverse probability of treatment weighting
OR: Odds ratio
PS: Propensity score
SD: Standard deviation
SDD: Standardized daily dose
