# Supplementary material for: Longitudinal patterns of antidepressant and benzodiazepine use associated with injurious falls in older adults with depression: a retrospective cohort study
Source: BMC Med. 2025 Aug 20;23:487. doi: 10.1186/s12916-025-04325-2 (PMC12366386; doi:10.1186/s12916-025-04325-2)
Supplement: Supplementary file 1 — Additional file 1: Table S1. The Strengthening the Reporting of Observational Studies in Epidemiology (STROBE) checklist. Table S2. Equivalency Conversion Table for Antidepressants and Benzodiazepines. Table S3. Nagin’s Diagnostic Criteria for Group-Based Multi-Trajectory Models of Antidepressant and Benzodiazepine Use among Medicare Beneficiaries (n=102,750). Table S4. International Classification of Diseases Clinical Modification Codes for Fall and Related Injuries. Table S5. International Classification of Diseases Clinical Modification Codes for Clinical Characteristics. Table S6. Detailed patient characteristics of eligible Medicare beneficiaries: Overall and by trajectory. Table S7. Trajectories of Antidepressant and Benzodiazepine Utilization Patterns and Risk of Falls and Related Injuries: Subgroup and Sensitivity analyses. Figure S1. Study Design Schematic Diagram. Figure S2. Cohort Selection Flowchart. [file 12916_2025_4325_MOESM1_ESM.docx]

ADDITIONAL FILES

Table S1. The Strengthening the Reporting of Observational Studies in Epidemiology (STROBE) checklist

Table S2. Equivalency Conversion Table for Antidepressants and Benzodiazepines

Table S3. Nagin’s Diagnostic Criteria for Group-Based Multi-Trajectory Models of Antidepressant and Benzodiazepine Use among Medicare Beneficiaries (n=102,750)

Table S4. International Classification of Diseases Clinical Modification Codes for Fall and Related Injuries

Table S5. International Classification of Diseases Clinical Modification Codes for Clinical Characteristics

Table S6. Detailed patient characteristics of eligible Medicare beneficiaries: Overall and by trajectory

Table S7. Trajectories of Antidepressant and Benzodiazepine Utilization Patterns and Risk of Falls and Related Injuries: Subgroup and Sensitivity analyses

Figure S1. Study Design Schematic Diagram

Figure S2. Cohort Selection Flowchart

**Table S1. The Strengthening the Reporting of Observational Studies in Epidemiology (STROBE) checklist**

|  | **Item No** | **Recommendation** | **Page/ lines** |
| --- | --- | --- | --- |
| **Title and abstract** | 1 | (*a*) Indicate the study’s design with a commonly used term in the title or the abstract | P.3, lines 40-42 |
|  |  | (*b*) Provide in the abstract an informative and balanced summary of what was done and what was found | P.3-4, lines 36-62 |
| **Introduction** | | |  |
| Background/rationale | 2 | Explain the scientific background and rationale for the investigation being reported | P.5, lines 67-89 |
| Objectives | 3 | State specific objectives, including any prespecified hypotheses | P.5-6, lines 89-91 |
| **Methods** | | |  |
| Study design | 4 | Present key elements of study design early in the paper | P.6-11, lines 93-240 |
| Setting | 5 | Describe the setting, locations, and relevant dates, including periods of recruitment, exposure, follow-up, and data collection | P.6-7, lines 96-123 |
| Participants | 6 | (*a*) Give the eligibility criteria, and the sources and methods of selection of participants. Describe methods of follow-up | P.6-7, lines 100-123 |
|  |  | (*b*) For matched studies, give matching criteria and number of exposed and unexposed | NA (no matching) |
| Variables | 7 | Clearly define all outcomes, exposures, predictors, potential confounders, and effect modifiers. Give diagnostic criteria, if applicable | P.7-9, lines 125-189 |
| Data sources/ measurement | 8* | For each variable of interest, give sources of data and details of methods of assessment (measurement). Describe comparability of assessment methods if there is more than one group | P.7-9, lines 125-189 |
| Bias | 9 | Describe any efforts to address potential sources of bias | P.9-10, lines 197-210 |
| Study size | 10 | Explain how the study size was arrived at | NA (population-based) |
| Quantitative variables | 11 | Explain how quantitative variables were handled in the analyses. If applicable, describe which groupings were chosen and why | P.10, lines 211-213 |
| Statistical methods | 12 | (*a*) Describe all statistical methods, including those used to control for confounding | P.9-10, lines 191-222 |
|  |  | (*b*) Describe any methods used to examine subgroups and interactions | P.11, lines 224-231 |
|  |  | (*c*) Explain how missing data were addressed | P.9, lines 191-192 |
|  |  | (*d*) If applicable, explain how loss to follow-up was addressed | P.10, lines 213-217 |
|  |  | (*e*) Describe any sensitivity analyses | P.11, lines 232-240 |
| **Results** | | |  |
| Participants | 13* | (a) Report numbers of individuals at each stage of study—eg numbers potentially eligible, examined for eligibility, confirmed eligible, included in the study, completing follow-up, and analysed | Additional files: Figure S2 |
|  |  | (b) Give reasons for non-participation at each stage | Additional files: Figure S2 |
|  |  | (c) Consider use of a flow diagram | Additional files: Figure S2 |
| Descriptive data | 14* | (a) Give characteristics of study participants (eg demographic, clinical, social) and information on exposures and potential confounders | Table 1, Additional files: Table S6 |
|  |  | (b) Indicate number of participants with missing data for each variable of interest | Missingness has been imputed |
|  |  | (c) Summarise follow-up time (eg, average and total amount) | Table 4 |
| Outcome data | 15* | Report numbers of outcome events or summary measures over time | Table 4 |
| Main results | 16 | (*a*) Give unadjusted estimates and, if applicable, confounder-adjusted estimates and their precision (eg, 95% confidence interval). Make clear which confounders were adjusted for and why they were included | Table 4 |
|  |  | (*b*) Report category boundaries when continuous variables were categorized | P.8, lines 164-171 |
|  |  | (*c*) If relevant, consider translating estimates of relative risk into absolute risk for a meaningful time period | Table 4 |
| Other analyses | 17 | Report other analyses done—eg analyses of subgroups and interactions, and sensitivity analyses | Additional files: Table S7 |
| **Discussion** | | |  |
| Key results | 18 | Summarise key results with reference to study objectives | P.13, lines 288-297 |
| Limitations | 19 | Discuss limitations of the study, taking into account sources of potential bias or imprecision. Discuss both direction and magnitude of any potential bias | P.16-18, lines 354-408 |
| Interpretation | 20 | Give a cautious overall interpretation of results considering objectives, limitations, multiplicity of analyses, results from similar studies, and other relevant evidence | P.14-16, lines 322-352 |
| Generalisability | 21 | Discuss the generalisability (external validity) of the study results | P.17, lines 378-383 |
| **Other information** | | |  |
| Funding | 22 | Give the source of funding and the role of the funders for the present study and, if applicable, for the original study on which the present article is based | P.20, lines 443-445 |

*Give information separately for exposed and unexposed groups.

**Table S2. Equivalency Conversion Table for Antidepressants and Benzodiazepines**

| **Antidepressants** | **Defined daily dose (mg)** | **Type (by mechanism of action)** |
| --- | --- | --- |
| Citalopram | 20 | SSRI |
| Escitalopram | 10 | SSRI |
| Fluoxetine | 20 | SSRI |
| Fluvoxamine | 100 | SSRI |
| Paroxetine | 20 | SSRI |
| Sertraline | 50 | SSRI |
| Desvenlafaxine | 50 | SNRI |
| Duloxetine | 60 | SNRI |
| Levomilnacipran | 40 | SNRI |
| Venlafaxine | 100 | SNRI |
| Isocarboxazid | 15 | MAOI |
| Phenelzine | 60 | MAOI |
| Tranylcypromine | 10 | MAOI |
| Bupropion | 300 | Others |
| Mirtazapine | 30 | Others |
| Nefazodone | 400 | Others |
| Trazodone | 300 | Others |
| Vortioxetine | 10 | Others |
| Esketamine | 8 | Others |
| **Benzodiazepines** | **Diazepam equivalent dose (mg)** | **Type (by half-life)** |
| Alprazolam | 0.5 | Short-acting |
| Midazolam | 7 | Short-acting |
| Oxazepam | 15 | Short-acting |
| Triazolam | 0.25 | Short-acting |
| Estazolam | 1 | Intermediate-acting |
| Lorazepam | 1 | Intermediate-acting |
| Temazepam | 10 | Intermediate-acting |
| Chlordiazepoxide | 10 | Long-acting |
| Clobazam | 20 | Long-acting |
| Clonazepam | 0.5 | Long-acting |
| Clorazepate | 7.5 | Long-acting |
| Diazepam | 10 | Long-acting |
| Flurazepam | 15 | Long-acting |

**Abbreviations**: MAOI: Monoamine Oxidase Inhibitors, SNRI: Serotonin-Norepinephrine Reuptake Inhibitors, SSRI: Selective Serotonin Reuptake Inhibitors

**Table S3. Nagin’s Diagnostic Criteria for Group-Based Multi-Trajectory Models of Antidepressant and Benzodiazepine Use among Medicare Beneficiaries (n=102,750)**

| **Trajectory Groups*** | **Number of Patients in Group** | **Model Estimate of Group Probability (95%CI)**^†^ | **Proportion Classified in Group**^‡^ | **Average Posterior Probability^§^** | **Odds Correct Classification^\|\|^** |
| --- | --- | --- | --- | --- | --- |
| A: low discontinuing AD | 17,820 | 17.3% (17.1, 17.6) | 17.3% | 99.9% | 71097128.01 |
| B: low declining AD | 31,824 | 30.6% (30.3, 30.9) | 31.0% | 98.8% | 1.49496E+14 |
| C: moderate increasing AD | 24,194 | 23.3% (23.0, 23.5) | 23.5% | 98.9% | 1.58022E+11 |
| D: high increasing AD | 5,586 | 5.4% (5.3, 5.5) | 5.4% | 99.4% | 4.50798E+15 |
| E: low discontinuing AD/very-low declining BZD | 4,161 | 4.0% (3.9, 4.2) | 4.0% | 99.6% | 1552924.982 |
| F: low discontinuing AD/low declining BZD | 717 | 0.7% (0.6, 0.7) | 0.7% | 97.9% | 9868301.012 |
| G: low declining AD/very-low declining BZD | 7,004 | 6.7% (6.5, 6.8) | 6.8% | 97.7% | 14817943977 |
| H: low declining AD/low declining BZD | 1,291 | 1.2% (1.1, 1.3) | 1.3% | 95.5% | 36071222086 |
| I: moderate increasing AD/very-low declining BZD | 6,351 | 6.0% (5.9, 6.2) | 6.2% | 97.6% | 1012412566 |
| J: moderate increasing AD/low stable BZD | 1,308 | 1.2% (1.2, 1.3) | 1.3% | 95.8% | 4432289144 |
| K: very-high increasing AD/very-low stable BZD | 1,771 | 1.7% (1.6, 1.8) | 1.7% | 98.3% | 7.17054E+15 |
| L: very-high increasing AD/low increasing BZD | 723 | 0.7% (0.6, 0.7) | 0.7% | 97.9% | 1.10073E+16 |

*To facilitate the labeling of AD and BZD dose levels for each trajectory, we defined AD use as: negligible (standardized daily dose [SDD] < 0.1 defined daily dose [DDD]), very low (0.1 to < 0.5 DDD), low (0.5 to <1 DDD), moderate (1 to <1.5 DDD), high (1.5 to <2 DDD) and very high dose (≥ 2 DDD). Similarly, we defined BZD use as negligible (SDD < 1 diazepam milligram equivalent [DME]), very-low (< 5 DME), low (5 to <10 DME), moderate (10 to <15 DME), high (15 to <20 DME) and very-high dose (≥20 DME). We defined a “discontinuing” pattern as a dose reduction to the negligible level, “declining” or “increasing” patterns when the absolute change exceeded 0.1 DDD for ADs or 1 DME for BZDs, and “stable” when changes remained below these thresholds.

^†^ 95% CIs, based on parametric bootstrap method, should be reasonably narrow.

^‡^ Proportion classified in group is based on the maximum posterior probability rule. The values of the proportion classified in the group should be similar to the model estimates of group probabilities in the third column.

^§^ Average posterior probability is calculated by averaging the posterior probabilities for a given group for all individuals included in this group by the maximum posterior probability rule. Acceptable values for this criterion are ≥0.7.

^||^ Acceptable values of the odds correct classification are ≥5.

Abbreviations: **AD**, antidepressant; **BZD,** benzodiazepine; **CI**, confidence interval

**Table S4. International Classification of Diseases Clinical Modification Codes for Fall and Related Injuries**

| **Type of FRI** | **ICD-9-CM** | **ICD-10-CM** |
| --- | --- | --- |
| Falls | E880, E8840, E8842, E8843, E8844, E8845, E8846, E8849, E8859, E887, E888 | W010XX, W0110X, W01110, W01118, W01119, W01190, W01198, W050XX, W051XX, W052XX, W06XXX, W07XXX, W08XXX, W100XX, W101XX, W102XX, W108XX, W109XX, W1781X, W1789X, W1800X, W1802X, W1809X, W1812X, W182XX, W1839X, W1849X, W19XXX |
| Head/Face Injuries | 800, 801, 802, 803, 804, 829, 850, 854, 8700, 8702, 8703, 8704, 8708, 8709, 8710, 8712, 8713, 8714, 8717, 8719, 87200, 87201, 87202, 87210, 87211, 87212, 87261, 87262, 87263, 87264, 87269, 8728, 8730, 8732, 8734, 8736, 87373, 8738, 9100, 9108, 9180, 9189, 920, 921, 9248, 9249, 830, 8398, 8399, 8480, 8481, 8482, 8488, 8489, 900, 925, 950, 951 | S0190, S020X, S0210, S0211, S0219, S022X, S0230, S0231, S0232, S0240, S0241, S0242, S0260, S0261, S0262, S0263, S0264, S0265, S0266, S0267, S0269, S0280, S0281, S0282, S0291, S0292, S060X, S061X, S062X, S0630, S0631, S0632, S0633, S0634, S0635, S0636, S0637, S0638, S064X, S065X, S066X, S0681, S0682, S0689, S069X, S0000, S0001, S0003, S0010, S0011, S0012, S0020, S0021, S0025, S0030, S0031, S0033, S0040, S0041, S0043, S0050, S0051, S0053, S0080, S0081, S0083, S0090, S0091, S0093, S0100, S0101, S0103, S0110, S0111, S0113, S0120, S0121, S0123, S0130, S0131, S0133, S0140, S0141, S0143, S0150, S0151, S0153, S0180, S0181, S0183, S0190, S0191, S0193, S025X, S032X, S0510, S0511, S0512, S0520, S0521, S0522, S0530, S0531, S0532, S0540, S0541, S0542, S0560, S0561, S0562, S058X, S0590, S0591, S0592, S080X, S0811, S0812, S0881, S0889, S0912, S0920, S0921, S0922, S0930, S0939, S098X, S0990, S0991, S0993, S0140, S0300, S0301, S0302, S0303, S038X, S039X, S0340, S0341, S0342, S0343, S038X, S039X, S0401, S0402, S0403, S0404, S0410, S0411, S0412, S0420, S0421, S0422, S0430, S0431, S0432, S0440, S0441, S0442, S0450, S0451, S0452, S0460, S0461, S0462, S0470, S0471, S0472, S0481, S0489, S049X, S070X, S071X, S078X, S079X, S090X, S1500, S1501, S1502, S1509, S1510, S1511, S1512, S1519, S1520, S1521, S1522, S1529, S1530, S1531, S1532, S1539, S158X, S159X |
| Neck/Trunk Injuries | 805, 806, 8070, 8071, 8072, 8073, 8074, 8075, 8076, 808, 809, 829, 8740, 8742, 8744, 8748, 8750, 8760, 8770, 8782, 8784, 8786, 8788, 8792, 8794, 8796, 8798, 9110, 9118, 9190, 9198, 922, 9248, 9249, 839, 846, 847, 8483, 84840, 84841, 84842, 84849, 8485, 8488, 8489, 860, 861, 862, 863, 864, 865, 866, 867, 868, 869, 9028, 926, 952, 953, 954 | S2231, S2232, S2239, S2241, S2242, S2243, S2249, S3230, S3231, S3239, S3240, S3241, S3242, S3243, S3244, S3245, S3246, S3247, S3248, S3249, S3250, S3251, S3259, S3260, S3261, S3269, S3281, S3282, S3289, S329X, S1200, S1201, S1202, S1203, S1204, S1209, S1210, S1211, S1212, S1213, S1214, S1215, S1219, S1220, S1223, S1224, S1225, S1229, S1230, S1233, S1234, S1235, S1239, S1240, S1243, S1244, S1245, S1249, S1250, S1253, S1254, S1255, S1259, S1260, S1263, S1264, S1265, S1269, S128X, S129X, S1410, S1411, S1412, S1413, S1415, S2200, S2201, S2202, S2203, S2204, S2205, S2206, S2207, S2208, S2220, S2221, S2222, S2223, S2224, S225X, S229X, S2410, S2411, S2413, S2415, S3200, S3201, S3202, S3203, S3204, S3205, S3210, S3211, S3212, S3213, S3214, S3215, S3216, S3217, S3219, S322X, S3410, S3411, S3412, S3413, S343X, S1080, S1081, S1083, S1090, S1091, S1093, S1101, S1102, S1111, S1121, S1180, S1181, S1189, S1190, S1191, S1193, S162X, S2000, S2001, S2002, S2010, S2011, S2014, S2020, S2021, S2022, S2030, S2031, S2040, S2041, S2090, S2091, S2110, S2111, S2113, S2120, S2121, S2123, S2190, S2191, S2193, S2902, S300X, S301X, S3081, S3091, S3092, S3100, S3101, S3103, S3110, S3111, S3113, S3180, S3181, S3182, S383X, S3902, M9910, M9911, M9912, M9913, M9914, M9915, M9916, M9917, M9918, M9919, S1190, S130X, S1310, S1311, S1312, S1313, S1314, S1315, S1316, S1317, S1318, S1320, S1329, S2110, S2120, S230X, S2310, S2311, S2312, S2313, S2314, S2315, S2316, S2317, S2320, S2329, S3100, S330X, S3310, S3311, S3312, S3313, S3314, S332X, S3330, S3339, S4320, S4321, S4322, S038X, S039X, S134X, S138X, S139X, S161X, S233X, S2341, S2342, S238X, S239X, S2901, S334X, S335X, S336X, S338X, S339X, S3901, S2600, S2601, S2602, S2609, S2610, S2611, S2612, S2619, S2690, S2691, S2692, S2699, S270X, S271X, S272X, S2730, S2732, S2733, S2739, S2740, S2742, S2749, S2750, S2753, S2759, S2760, S2763, S2769, S2780, S2789, S279X, S3101, S3103, S3160, S3161, S3163, S3600, S3602, S3603, S3609, S3611, S3612, S3620, S3622, S3623, S3624, S3625, S3626, S3629, S3630, S3689, S3690, S3692, S3693, S3699, S3700, S3701, S3702, S3703, S3704, S3705, S3706, S3709, S3719, S3729, S3781, S140X, S1410, S1411, S1412, S1413, S1414, S1415, S142X, S143X, S145X, S170X, S178X, S179X, S240X, S2410, S2411, S2413, S2414, S2415, S242X, S243X, S244X, S248X, S249X, S280X, S3401, S3402, S3410, S3411, S3412, S3413, S3421, S3422, S343X, S344X, S345X, S346X, S348X, S349X, S358X |
| Upper Extremity Injuries | 810, 811, 812, 813, 814, 815, 816, 817, 818, 819, 829, 88000, 88001, 88002, 88003, 88009, 88020, 88021, 88022, 88023, 88029, 88100, 88101, 88102, 88110, 88120, 88121, 88122, 8820, 8822, 8830, 8832, 8840, 8842, 9120, 9128, 9130, 9138, 9140, 9148, 9150, 9158, 92300, 92301, 92302, 92303, 92309, 92310, 92311, 92320, 92321, 9233, 9238, 9239, 9248, 9249, 831, 832, 833, 834, 8398, 8399, 841, 8489, 840, 8420, 8421, 903, 927, 955 | S4200, S4201, S4202, S4203, S4220, S4221, S4222, S4223, S4224, S4225, S4226, S4227, S4229, S4240, S4241, S4242, S4243, S4244, S4245, S4246, S4247, S4248, S4249, S4900, S4901, S4902, S4903, S4904, S4909, S4910, S4911, S4912, S4913, S4914, S4919, S4230, S4231, S4232, S4233, S4234, S4235, S4236, S4239, S4290, S4291, S4292, S5200, S5201, S5202, S5203, S5204, S5209, S5210, S5211, S5212, S5213, S5218, S5220, S5221, S5222, S5223, S5224, S5225, S5226, S5227, S5228, S5229, S5230, S5231, S5232, S5233, S5234, S5235, S5236, S5237, S5238, S5239, S5250, S5251, S5252, S5253, S5254, S5255, S5256, S5257, S5259, S5260, S5261, S5262, S5269, S5290, S5291, S5292, S5900, S5901, S5902, S5903, S5904, S5909, S5910, S5911, S5912, S5913, S5914, S5919, S5920, S5921, S5922, S5923, S5924, S5929, S6200, S6201, S6202, S6203, S6210, S6215, S6220, S6221, S6222, S6223, S6224, S6225, S6229, S6230, S6231, S6232, S6233, S6234, S6235, S6236, S6239, S6250, S6251, S6252, S6260, S6261, S6262, S6263, S6264, S6265, S6266, S6290, S6291, S6292, S4210, S4211, S4212, S4213, S4214, S4215, S4219, S6211, S6212, S6213, S6214, S6216, S6217, S6218, S4100, S4101, S4103, S4110, S4111, S4113, S4602, S4612, S4622, S4632, S4682, S4692, S5100, S5101, S5103, S5180, S5181, S5183, S5602, S5612, S5622, S5632, S5642, S5652, S5682, S5692, S6100, S6101, S6103, S6110, S6111, S6113, S6120, S6121, S6123, S6130, S6131, S6133, S6140, S6141, S6143, S6150, S6151, S6153, S6602, S6612, S6622, S6632, S6642, S6652, S6682, S6692, S4021, S4081, S4091, S4092, S5031, S5081, S5090, S5091, S6031, S6039, S6041, S6051, S6081, S6091, S6092, S6093, S6094, S4001, S4002, S5000, S5001, S5002, S5010, S5011, S5012, S6000, S6001, S6002, S6003, S6004, S6005, S6010, S6011, S6012, S6013, S6014, S6015, S6021, S6022, S4100, S4110, S4300, S4301, S4302, S4303, S4308, S4310, S4311, S4312, S4313, S4314, S4315, S4330, S4331, S4339, S5100, S5300, S5301, S5302, S5303, S5309, S5310, S5311, S5312, S5313, S5314, S5319, S6100, S6120, S6140, S6310, S6311, S6312, S6320, S6321, S6322, S6323, S6324, S6325, S6326, S6327, S6328, S6329, S4340, S4341, S4342, S4343, S4349, S4350, S4351, S4352, S4360, S4361, S4362, S4380, S4381, S4382, S4390, S4391, S4392, S4601, S4611, S4621, S4631, S4681, S4691, S6330, S6331, S6332, S6333, S6339, S6350, S6351, S6352, S6359, S6601, S6611, S6621, S6631, S6641, S6651, S6681, S6691, S6340, S6341, S6342, S6343, S6349, S6360, S6361, S6362, S6363, S6364, S6365, S6368, S6369, S638X, S6390, S6391, S6392, S4400, S4401, S4402, S4410, S4411, S4412, S4420, S4421, S4422, S4430, S4431, S4432, S4440, S4441, S4442, S4450, S4451, S4452, S448X, S4490, S4491, S4492, S4500, S4501, S4509, S4510, S4511, S4519, S4520, S4521, S4529, S4530, S4531, S4539, S4580, S4581, S4589, S4590, S4591, S4599, S471X, S472X, S479X, S5400, S5401, S5402, S5410, S5411, S5412, S5420, S5421, S5422, S5430, S5431, S5432, S548X, S5490, S5491, S5492, S5500, S5501, S5509, S5510, S5511, S5519, S5520, S5521, S5529, S5580, S5581, S5589, S5590, S5591, S5599, S5700, S5701, S5702, S5780, S5781, S5782, S6400, S6401, S6402, S6410, S6411, S6412, S6420, S6421, S6422, S6430, S6431, S6432, S6440, S6449, S648X, S6490, S6491, S6492, S6500, S6501, S6509, S6510, S6511, S6519, S6520, S6521, S6529, S6530, S6531, S6539, S6540, S6541, S6549, S6550, S6551, S6559, S6580, S6581, S6589, S6590, S6591, S6599, S6700, S6701, S6702, S6710, S6719, S6720, S6721, S6722, S6730, S6731, S6732, S6740, S6741, S6742, S6790, S6791, S6792 |
| Lower Extremity Injuries | 820, 821, 822, 823, 824, 825, 826, 827, 828, 829, 924, 8910, 8912, 9160, 9168, 9170, 9178, 835, 904, 928, 956 | S7200, S7201, S7202, S7203, S7204, S7205, S7206, S7209, S7210, S7211, S7212, S7213, S7214, S7221, S7222, S7223, S7224, S7225, S7226, S7900, S7901, S7909, S7230, S7232, S7233, S7234, S7235, S7236, S7239, S7240, S7241, S7242, S7243, S7244, S7245, S7246, S7247, S7249, S728X, S7290, S7291, S7292, S7910, S7911, S7912, S7913, S7914, S7919, S8210, S8211, S8212, S8213, S8214, S8215, S8216, S8219, S8220, S8222, S8223, S8224, S8225, S8226, S8229, S8231, S8240, S8242, S8243, S8244, S8245, S8246, S8249, S8281, S8282, S8283, S8286, S8900, S8901, S8902, S8903, S8904, S8909, S8920, S8921, S8922, S8929, S8200, S8201, S8202, S8203, S8204, S8209, S8230, S8239, S8251, S8252, S8253, S8254, S8255, S8256, S8261, S8262, S8263, S8264, S8265, S8266, S8284, S8285, S8287, S8289, S8910, S8911, S8912, S8913, S8914, S8919, S8930, S8931, S8932, S8939, S8290, S8291, S8292, S9200, S9201, S9202, S9203, S9204, S9205, S9206, S9210, S9211, S9212, S9213, S9214, S9215, S9219, S9220, S9221, S9222, S9223, S9224, S9225, S9230, S9231, S9232, S9233, S9234, S9235, S9240, S9241, S9242, S9249, S9250, S9251, S9252, S9253, S9259, S9281, S9290, S9291, S9900, S9901, S9902, S9903, S9904, S9909, S9910, S9911, S9912, S9913, S9914, S9919, S9920, S9921, S9922, S9923, S9924, S9929, S7100, S7101, S7103, S7110, S7111, S7113, S7602, S7612, S7622, S7632, S7682, S7692, S8100, S8101, S8103, S8180, S8181, S8183, S8602, S8612, S8622, S8632, S8682, S8692, S9100, S9101, S9103, S9602, S9612, S9622, S9682, S9692, S9130, S9131, S9133, S9110, S9111, S9113, S9120, S9121, S9123, S7021, S7031, S7091, S7092, S8021, S8081, S8091, S8092, S9041, S9051, S9081, S9091, S9092, S9093, S7000, S7001, S7002, S7010, S7011, S7012, S8000, S8001, S8002, S8010, S8011, S8012, S9000, S9001, S9002, S9011, S9012, S9021, S9022, S9030, S9031, S9032, S7100, S7300, S7301, S7302, S7303, S7304, S7400, S7401, S7402, S7410, S7411, S7412, S7420, S7421, S7422, S748X, S7490, S7491, S7492, S7500, S7501, S7502, S7509, S7510, S7511, S7512, S7519, S7520, S7521, S7522, S7529, S7580, S7581, S7589, S7590, S7591, S7599, S7700, S7701, S7702, S7710, S7711, S7712, S7720, S7721, S7722, S8400, S8401, S8402, S8410, S8411, S8412, S8420, S8421, S8422, S8480, S8490, S8491, S8492, S8500, S8501, S8509, S8510, S8511, S8512, S8513, S8514, S8515, S8516, S8517, S8518, S8520, S8521, S8529, S8530, S8531, S8539, S8540, S8541, S8549, S8550, S8551, S8559, S8580, S8581, S8589, S8590, S8591, S8599, S8700, S8701, S8702, S8780, S8781, S8782, S9400, S9401, S9402, S9410, S9411, S9412, S9420, S9421, S9422, S9430, S9431, S9432, S948X, S9490, S9491, S9492, S9500, S9501, S9509, S9510, S9511, S9519, S9520, S9521, S9529, S9580, S9581, S9589, S9590, S9591, S9599, S9700, S9701, S9702, S9710, S9711, S9712, S9780, S9781, S9782 |
| Fractures | 800, 801, 802, 803, 804, 829, 850, 854, 805, 806, 8070, 8071, 8072, 8073, 8074, 8075, 8076, 808, 809, 829, 810, 811, 812, 813, 814, 815, 816, 817, 818, 819, 829, 820, 821, 822, 823, 824, 825, 826, 827, 828, 829 | S0190, S020X, S0210, S0211, S0219, S022X, S0230, S0231, S0232, S0240, S0241, S0242, S0260, S0261, S0262, S0263, S0264, S0265, S0266, S0267, S0269, S0280, S0281, S0282, S0291, S0292, S060X, S061X, S062X, S0630, S0631, S0632, S0633, S0634, S0635, S0636, S0637, S0638, S064X, S065X, S066X, S0681, S0682, S0689, S069X, S2231, S2232, S2239, S2241, S2242, S2243, S2249, S3230, S3231, S3239, S3240, S3241, S3242, S3243, S3244, S3245, S3246, S3247, S3248, S3249, S3250, S3251, S3259, S3260, S3261, S3269, S3281, S3282, S3289, S329X, S1200, S1201, S1202, S1203, S1204, S1209, S1210, S1211, S1212, S1213, S1214, S1215, S1219, S1220, S1223, S1224, S1225, S1229, S1230, S1233, S1234, S1235, S1239, S1240, S1243, S1244, S1245, S1249, S1250, S1253, S1254, S1255, S1259, S1260, S1263, S1264, S1265, S1269, S128X, S129X, S1410, S1411, S1412, S1413, S1415, S2200, S2201, S2202, S2203, S2204, S2205, S2206, S2207, S2208, S2220, S2221, S2222, S2223, S2224, S225X, S229X, S2410, S2411, S2413, S2415, S3200, S3201, S3202, S3203, S3204, S3205, S3210, S3211, S3212, S3213, S3214, S3215, S3216, S3217, S3219, S322X, S3410, S3411, S3412, S3413, S343X, S4200, S4201, S4202, S4203, S4220, S4221, S4222, S4223, S4224, S4225, S4226, S4227, S4229, S4240, S4241, S4242, S4243, S4244, S4245, S4246, S4247, S4248, S4249, S4900, S4901, S4902, S4903, S4904, S4909, S4910, S4911, S4912, S4913, S4914, S4919, S4230, S4231, S4232, S4233, S4234, S4235, S4236, S4239, S4290, S4291, S4292, S5200, S5201, S5202, S5203, S5204, S5209, S5210, S5211, S5212, S5213, S5218, S5220, S5221, S5222, S5223, S5224, S5225, S5226, S5227, S5228, S5229, S5230, S5231, S5232, S5233, S5234, S5235, S5236, S5237, S5238, S5239, S5250, S5251, S5252, S5253, S5254, S5255, S5256, S5257, S5259, S5260, S5261, S5262, S5269, S5290, S5291, S5292, S5900, S5901, S5902, S5903, S5904, S5909, S5910, S5911, S5912, S5913, S5914, S5919, S5920, S5921, S5922, S5923, S5924, S5929, S6200, S6201, S6202, S6203, S6210, S6215, S6220, S6221, S6222, S6223, S6224, S6225, S6229, S6230, S6231, S6232, S6233, S6234, S6235, S6236, S6239, S6250, S6251, S6252, S6260, S6261, S6262, S6263, S6264, S6265, S6266, S6290, S6291, S6292, S4210, S4211, S4212, S4213, S4214, S4215, S4219, S6211, S6212, S6213, S6214, S6216, S6217, S6218, S7200, S7201, S7202, S7203, S7204, S7205, S7206, S7209, S7210, S7211, S7212, S7213, S7214, S7221, S7222, S7223, S7224, S7225, S7226, S7900, S7901, S7909, S7230, S7232, S7233, S7234, S7235, S7236, S7239, S7240, S7241, S7242, S7243, S7244, S7245, S7246, S7247, S7249, S728X, S7290, S7291, S7292, S7910, S7911, S7912, S7913, S7914, S7919, S8210, S8211, S8212, S8213, S8214, S8215, S8216, S8219, S8220, S8222, S8223, S8224, S8225, S8226, S8229, S8231, S8240, S8242, S8243, S8244, S8245, S8246, S8249, S8281, S8282, S8283, S8286, S8900, S8901, S8902, S8903, S8904, S8909, S8920, S8921, S8922, S8929, S8200, S8201, S8202, S8203, S8204, S8209, S8230, S8239, S8251, S8252, S8253, S8254, S8255, S8256, S8261, S8262, S8263, S8264, S8265, S8266, S8284, S8285, S8287, S8289, S8910, S8911, S8912, S8913, S8914, S8919, S8930, S8931, S8932, S8939, S8290, S8291, S8292, S9200, S9201, S9202, S9203, S9204, S9205, S9206, S9210, S9211, S9212, S9213, S9214, S9215, S9219, S9220, S9221, S9222, S9223, S9224, S9225, S9230, S9231, S9232, S9233, S9234, S9235, S9240, S9241, S9242, S9249, S9250, S9251, S9252, S9253, S9259, S9281, S9290, S9291, S9900, S9901, S9902, S9903, S9904, S9909, S9910, S9911, S9912, S9913, S9914, S9919, S9920, S9921, S9922, S9923, S9924, S9929 |
| **Exclusion** |  |  |
| Bone Cancer | 1700, 1701, 1702, 1703, 1704, 1705, 1706, 1707, 1708, 1709, 1960, 1961, 1962, 1963, 1965, 1966, 1968, 1969, 1970, 1971, 1972, 1973, 1974, 1975, 1976, 1977, 1978, 1980, 1981, 1982, 1983, 1984, 1985, 1986, 1987, 19881, 19882, 19889, 7331 | C400, C401, C410, C411, C412, C413, C414, C418, C402, C403, C409, C408, C419, C7951, C7952 |
| Motor vehicle accidents | E810, E811, E812, E813, E814, E815, E816, E817, E818, E819, E820, E821, E822, E823, E824, E825, E826, E827, E828, E829, E830, E831, E832, E833, E834, E835, E836, E837, E838, E839, E840, E841, E842, E843, E844, E845, E846, E847, E848, E849 | V00, V01, V02, V03, V04, V05, V06, V07, V08, V09, V10, V11, V12, V13, V14, V15, V16, V17, V18, V19, V20, V21, V22, V23, V24, V25, V26, V27, V28, V29, V30, V31, V32, V33, V34, V35, V36, V37, V38, V39, V40, V41, V42, V43, V44, V45, V46, V47, V48, V49, V50, V51, V52, V53, V54, V55, V56, V57, V58, V59, V60, V61, V62, V63, V64, V65, V66, V67, V68, V69, V70, V71, V72, V73, V74, V75, V76, V77, V78, V79, V80, V81, V82, V83, V84, V85, V86, V87, V88, V89, V90, V91, V92, V93, V94, V95, V96, V97, V98, V99 |
| Contact with other objects, gunshots, drowning, electrocutions | E910, E916, E917, E918, E919, E920, E921, E922, E923, E925, E926, E928, E929 | W20, W21, W22, W23, W24, W25, W26, W27, W28, W29, W30, W31, W32, W33, W34, W35, W36, W37, W38, W39, W40, W41, W42, W43, W44, W45, W46, W47, W48, W49, W50, W51, W52, W53, W54, W55, W56, W57, W58, W59, W60, W61, W62, W63, W64, W65, W66, W67, W68, W69, W70, W71, W72, W73, W74, W75, W76, W77, W78, W79, W80, W81, W82, W83, W84, W85, W86, W87, W88, W89, W90, W91, W92, W93, W94, W95, W96, W97, W98, W99 |
| Burns and exposure | E890, E891, E892, E893, E894, E895, E896, E897, E898, E899, E924 | X00, X01, X02, X03, X04, X05, X06, X07, X08, X09, X10, X11, X12, X13, X14, X15, X16, X17, X18, X19, X20, X21, X22, X23, X24, X25, X26, X27, X28, X29, X30, X31, X32, X33, X34, X35, X36, X37, X38, X39, X40, X41, X42, X43, X44, X45, X46, X47, X48, X49, X50, X51, X52, X53, X54, X55, X56, X57, X58, X59, X60, X61, X62, X63, X64, X65, X66, X67, X68, X69, X70, X71, X72, X73, X74, X75, X76, X77, X78, X79, X80, X81, X82, X83, X84, X85, X86, X87, X88, X89, X90, X91, X92, X93, X94, X95, X96, X97, X98, X99 |
| Self-harm | E950, E951, E952, E953, E954, E955, E956, E957, E958, E959 | Y00, Y01, Y02, Y03, Y04, Y05, Y06, Y07, Y08, Y09, Y10, Y11, Y12, Y13, Y14, Y15, Y16, Y17, Y18, Y19, Y20, Y21, Y22, Y23, Y24, Y25, Y26, Y27, Y28, Y29, Y30, Y31, Y32, Y33, Y34, Y35, Y36, Y37, Y38, Y39, Y40, Y41, Y42, Y43, Y44, Y45, Y46, Y47, Y48, Y49, Y50, Y51, Y52, Y53, Y54, Y55, Y56, Y57, Y58, Y59, Y60, Y61, Y62, Y63, Y64, Y65, Y66, Y67, Y68, Y69, Y70, Y71, Y72, Y73, Y74, Y75, Y76, Y77, Y78, Y79, Y80, Y81, Y82, Y83, Y84, Y85, Y86, Y87, Y88, Y89, Y90, Y91, Y92, Y93, Y94, Y95, Y96, Y97, Y98, Y99 |

**Table S5. International Classification of Diseases Clinical Modification Codes for Clinical Characteristics**

| **Clinical characteristics** | **ICD-9-CM** | **ICD-10-CM** |
| --- | --- | --- |
| Alcohol abuse/dependence | 291, 303, 3050 | F101, F102 |
| Anxiety disorder | 29384, 3000, 30010, 3002, 3003, 3005, 30089, 3009, 308, 30981, 3130, 3131, 31321, 31322, 3133, 31382, 31383 | F430, R457, F41, F40, F42, F488, F489, F99, F938, F064, F449, F431 |
| Bipolar disorder | 2960, 2961, 2964, 2965, 2966, 2967, 2968, 2969 | F30, F31 |
| Chronic pain |  |  |
| Abdominal pain/hernia | 541, 5409, 5531, 5533, 5641, 5770, 53390, 53500, 55090, 55092, 55320, 55321, 55329, 59080, 78900, 78901, 78902, 78903, 78904, 78905, 78906, 78907, 78909 | K37, K3580, K3589, K429, K449, K581, K582, K588, K589, K8590, K8591, K8592, K279, K2900, K4090, K4020, K439, K432, K469, N12, R109, R1011, R1012, R1031, R1032, R1033, R1013, R1084, R1010, R102, R1030 |
| Back pain | 720, 724, 846, 7214, 7215, 7216, 7217, 7218, 7221, 7222, 7226, 7371, 7372, 7373, 7384, 7385, 7392, 7393, 7394, 8054, 8056, 8058, 8392, 8471, 8472, 8473, 8479, 72230, 72232, 72252, 72270, 72272, 72273, 72280, 72282, 72283, 72290, 72292, 72293, 75610, 75611, 75612, 75613, 75614, 75615, 75616, 75617, 75619, 83942 | M4000, M40209, M40299, M404, M405, M4100, M4120, M4130, M4180, M419, M4300, M4310, M4327, M4328, M4389, M459, M4600, M461, M4640, M4645, M4647, M465, M468, M469, M4714, M4715, M4716, M4800, M4804, M4806, M4808, M481, M482, M483, M489, M498, M5104, M5105, M5106, M512, M513, M5136, M5137, M5146, M5147, M518, M519, M5327, M5328, M533, M539, M5408, M5414, M5415, M5416, M5417, M5430, M545, M546, M5489, M549, M961, M962, M963, M964, M965, M9902, M9903, M9904, M9983, M9984, Q760, Q761, Q762, Q76419, Q7649, S129A, S22009A, S23101A, S233A, S238A, S239A, S32009A, S3210A, S322A, S33101A, S332A, S335A, S336A, S338A, S339A |
| Catastrophizing | 30780, 30789 | F454 |
| Chest pain | 4139, 78650, 78651, 78652, 78659 | I208, I209, R079, R072, R071, R0781, R0782, R0789 |
| Internal orthopedic device implant and graft | 9964 | M979A, T84019A, T84029A, T84039A, T84059A, T84069A, T84099A, T84119A, T84129A, T84199A, T84498A |
| Fibromyalgia | 7291 | M609, M791, M797 |
| Headache/migraine | 346, 7840, 30781 | G43019, G43109, G43119, G43711, G43809, G43819, G43909, G43919, G43A, G43B, G43C, G43D, G441, R51, G44209 |
| Injury | 920, 8020, 8052, 8072, 8088, 8208, 8220, 8240, 8242, 8244, 8246, 8248, 8250, 8260, 8290, 8509, 8730, 8798, 8820, 8832, 8860, 8910, 8920, 8930, 9100, 9130, 9140, 9160, 9190, 9221, 9222, 9233, 9239, 9243, 9245, 9248, 9249, 9273, 9592, 9597, 9599, 73313, 80700, 80701, 81000, 81200, 81209, 81220, 81240, 81301, 81305, 81341, 81381, 81400, 81401, 81500, 81600, 81602, 81610, 82300, 82380, 82381, 82520, 82525, 83104, 87343, 88100, 88101, 92231, 92232, 92300, 92310, 92311, 92320, 92321, 92400, 92401, 92410, 92411, 92420, 92421, 95901, 95911, 95912, 95913, 95914, 95919, E8859, E887, E8880, E8881, E8888, E8889, E9060, E9063 | M4850A, M8008A, M8448A, M8468A, S0001A , S0031A, S00419A, S00511A , S00512A , S0091A , S0093A, S0100A, S01501A, S022A, S0600A, S098A, S0990A , S1011A , S1091A, S1093A, S20219A , S22009A, S2220A , S2239A, S298A, S300A, S300A , S301A, S31000A, S329A, S3981A, S3982A , S39840A, S39848A, S40019A, S40019A , S42009A, S42209A , S42293A, S42296A, S42309A, S42409A, S43109A, S4980A , S4990A, S5000A, S5010A, S50319A, S50819A , S51009A , S51809A , S52023A, S52026A , S52123A , S52126A, S52539A, S52549A, S5290A, S6000A , S60019A , S6010A, S60219A, S60229A, S60519A, S60819A, S61109A, S61209A, S61409A , S62009A, S62109A , S62309A, S62509A, S62509B , S62523A, S62526A , S62609A , S62609B, S62639A , S62669A, S66529A, S6700A, S6710A, S68119A, S68129A, S68619A, S68629A, S7000A, S7010A, S70219A , S70319A, S72009A , S8000A , S8010A , S80819A, S81009A, S81809A , S82009A, S82109A, S82201A, S82401A, S8253A, S8256A , S8263A, S8266A, S82843A, S82846A , S82853A , S82856A, S82899A , S8980A, S8990A , S9000A, S90119A, S90129A, S90229A, S9030A, S90519A, S91009A, S91109A , S91309A, S92009A, S92309A, S92403A, S92406A , S92503A, S92506A , S92819A , S92909A, S99009A, S99019A , S99029A , S99039A, S99049A, S99099A , S99109A, S99119A , S99129A, S99139A, S99149A , S99199A , S99819A , S99919A, T07A , T148A, T148A , T1490A, W01110A, W01198A, W1830A , W1849A, W19A, W540A , W5501A |
| Neck pain | 723, 7210, 7211, 7220, 7224, 8390, 8391, 8470, 72231, 72271, 72281, 72291 | M436, M4712, M47812, M4802, M5000, M5020, M5030, M5080, M5090, M5144, M5145, M530, M531, M5382, M5402, M5412, M5413, M542, M6788, M961, S1190A, S13101A, S13111A, S13121A, S13131A, S13141A, S13151A, S13161A, S13171A, S13181A, S134A, S138A |
| Osteoarthritis | 711, 712, 713, 730, 731, 732, 733, 734, 735, 736, 737, 738, 739, 7151, 7152, 7153, 7158, 7159, 7161, 7212, 7213, 7219, 7270, 71500, 71504, 71509 | D481, M0000, M00019, M00029, M0010, M00119, M00129, M0020, M00219, M00229, M0080, M00819, M00829, M009, M020, M118, M125, M1480, M150, M151, M152, M153, M158, M159, M1610, M167, M169, M1710, M175, M179, M189, M190, M192, M1990, M1991, M1993, M200, M201, M202, M203, M204, M205, M206, M21, M214, M40, M41, M42 , M430, M431, M438, M462, M463, M47814, M47817, M47819, M48, M651, M653, M654, M658, M6580, M659, M80, M81, M84, M85, M861, M862, M866, M869, M87, M88, M89, M8938, M894, M896, M8988, M905, M906, M908, M91, M92, M93, M94, M95, M962, M963, M964, M99, M998, M999, S02, S12, S22, S32, S42, S49, S52, S62, S68, S72, S82, S92 |
| Menstrual/genital reproductive pain | 6253, 6258, 6259, 6266, 6271, 6272 | N946, N9489, R102, N921, N950, N951 |
| Rheumatoid arthritis | 714 | M05, M06, M080, M082, M083, M088, M089, M120 |
| Kidney/gall bladder stones | 5920, 5921, 5929, 5941, 57420, 57510 | N200, N201, N209, N210, K8018, K8020, K819 |
| Temporomandibular disorder | 52460, 52461, 52462, 52463, 52469 | M26601, M26602, M26603, M26609, M26611, M26612, M26613, M26619, M26621, M26622, M26623, M26629, M26631, M26632, M26633, M26639, M2669 |
| Others | 470, 725, 726, 727, 728, 729, 3829, 5224, 5225, 5259, 5651, 7030, 7062, 8483, 8488, 8489, 37991, 38022, 38023, 38181, 38870, 56942, 60490, 61171, 61179, 71930, 78652, 87363 | M6747, M7030, D481, H5713, H60509, H60519 , H60529 , H60539, H60549 , H60559 , H60599, H6060, H6081 , H6090, H6690, H6980, H9209, J342 , K044, K047, K089, K603, K604, K605, K6289, L600 , L723 , M1230, M1240 , M2010, M2161 , M2162 , M2420 , M2570, M25729 , M353, M357, M5410, M60009 , M6010 , M6020, M609, M6100, M6110 , M6140 , M6159 , M619, M6200 , M6210, M623, M6240, M6250 , M62838 , M6284, M6289, M629 , M6500, M6520, M6530 , M654 , M6580, M65849 , M65879, M659 , M6610, M6618 , M66239, M66249 , M66259, M66269, M66339 , M66349, M66369 , M66829, M66879, M6688 , M669 , M6700, M6740, M6741, M6742 , M6743 , M6744, M6745 , M6746 , M6750, M6780, M6788 , M6790 , M70039 , M701, M702, M703, M7040, M705, M706, M707, M7098 , M7100 , M7120 , M7130, M7140 , M7150, M7180, M719 , M720 , M721 , M722 , M724, M726 , M729, M750, M7510, M75120, M752, M753, M754, M755, M758, M7610 , M7620, M7640 , M7650, M7660 , M76829 , M76899, M7700, M7710 , M7720 , M7730, M7740 , M7750, M778 , M779 , M790, M791 , M792, M793 , M794, M795 , M79609, M797, M7981, M7989, M799, N451, N452, N453 , N644 , N6451, N6452, N6453, N6459, R071, R0781, R252, R29898 , S025A, S025B, S039A, S2341A , S29019A, S39011A , T1490A |
| Congestive heart failure | 39891, 428 | I0981, I501, I5020, I5021, I5022, I5023, I5030, I5031, I5032, I5033, I5040, I5041, I5042, I5043, I50810, I50811, I50812, I50813, I50814, I5082, I5083, I5084, I5089, I509, I5181, I97130, I97131, O29121, O29122, O29123, O29129, R570, Z95811, Z95812 |
| Dementia | 290, 2911, 2912, 29282, 294, 331, 797 | F0150, F0151, F0152, F0153, F0154, F01A0, F01A11, F01A18, F01A2, F01A3, F01A4, F01B0, F01B11, F01B18, F01B2, F01B3, F01B4, F01C0, F01C11, F01C18, F01C2, F01C3, F01C4, F0280, F0281, F0282, F0283, F0284, F02A0, F02A11, F02A18, F02A2, F02A3, F02A4, F02B0, F02B11, F02B18, F02B2, F02B3, F02B4, F02C0, F02C11, F02C18, F02C2, F02C3, F02C4, F0390, F0391, F0392, F0393, F0394, F03A0, F03A11, F03A18, F03A2, F03A3, F03A4, F03B0, F03B11, F03B18, F03B2, F03B3, F03B4, F03C0, F03C11, F03C18, F03C2, F03C3, F03C4, F0670, F0671, G300, G301, G308, G309, G3101, G3109, G311, G312, G3181, G3182, G3183, G3185, G3189, G319 |
| Diabetes | 25000-25033, 64800-64804, 24900-24931, 25040-25093, 7751, 24940-24991 | E0800, E0801, E0810, E0811, E089, E0900, E0901, E0910, E0911, E099, E1010, E1011, E109, E1100, E1101, E1110, E1111, E119, E1300, E1301, E1310, E1311, E139, O24011, O24012, O24013, O24019, O2402, O2403, O24111, O24112, O24113, O24119, O2412, O2413, O24311, O24312, O24313, O24319, O2432, O2433, O24410, O24414, O24415, O24419, O24420, O24424, O24425, O24429, O24430, O24434, O24435, O24439, O24811, O24812, O24813, O24819, O2482, O2483, O24911, O24912, O24913, O24919, O2492, O2493, E0821, E0822, E0829, E08311, E08319, E08321, E08329, E08331, E08339, E08341, E08349, E08351, E083521, E083522, E083523, E083529, E083531, E083532, E083533, E083539, E083541, E083542, E083543, E083549, E083551, E083552, E083553, E083559, E08359, E0836, E0837X1, E0837X2, E0837X3, E0837X9, E0839, E0840, E0841, E0842, E0843, E0844, E0849, E0851, E0852, E0859, E08610, E08618, E08620, E08621, E08622, E08628, E08630, E08638, E08641, E08649, E0865, E0869, E088, E0921, E0922, E0929, E09311, E09319, E09321, E09329, E09331, E09339, E09341, E09349, E09351, E093521, E093522, E093523, E093529, E093531, E093532, E093533, E093539, E093541, E093542, E093543, E093549, E093551, E093552, E093553, E093559, E09359, E0936, E0937X1, E0937X2, E0937X3, E0937X9, E0939, E0940, E0941, E0942, E0943, E0944, E0949, E0951, E0952, E0959, E09610, E09618, E09620, E09621, E09622, E09628, E09630, E09638, E09641, E09649, E0965, E0969, E098, E1021, E1022, E1029, E10311, E10319, E10321, E10329, E10331, E10339, E10341, E10349, E103491, E10351, E103521, E103522, E103523, E103529, E103531, E103532, E103533, E103539, E103541, E103542, E103543, E103549, E103551, E103552, E103553, E103559, E10359, E1036, E1037X1, E1037X2, E1037X3, E1037X9, E1039, E1040, E1041, E1042, E1043, E1044, E1049, E1051, E1052, E1059, E10610, E10618, E10620, E10621, E10622, E10628, E10630, E10638, E10641, E10649, E1065, E1069, E108, E1121, E1122, E1129, E11311, E11319, E11321, E11329, E11331, E11339, E11341, E11349, E11351, E113521, E113522, E113523, E113529, E113531, E113532, E113533, E113539, E113541, E113542, E113543, E113549, E113551, E113552, E113553, E113559, E11359, E1136, E1137X1, E1137X2, E1137X3, E1137X9, E1139, E1140, E1141, E1142, E1143, E1144, E1149, E1151, E1152, E1159, E11610, E11618, E11620, E11621, E11622, E11628, E11630, E11638, E11641, E11649, E1165, E1169, E118, E1321, E1322, E1329, E13311, E13319, E13321, E13329, E13331, E13339, E13341, E13349, E13351, E133521, E133522, E133523, E133529, E133531, E133532, E133533, E133539, E133541, E133542, E133543, E133549, E133551, E133552, E133553, E133559, E13359, E1336, E1337X1, E1337X2, E1337X3, E1337X9, E1339, E1340, E1341, E1342, E1343, E1344, E1349, E1351, E1352, E1359, E13610, E13618, E13620, E13621, E13622, E13628, E13630, E13638, E13641, E13649, E1365, E1369, E138 |
| Drug abuse/dependence | 2920, 29282-29289, 2929, 30400-30493, 64830-64834 | F1110, F1111, F11120, F11121, F11122, F11129, F1113, F1114, F11150, F11151, F11159, F11181, F11182, F11188, F1119, F1120, F1121, F11220, F11221, F11222, F11229, F1123, F1124, F11250, F11251, F11259, F11281, F11282, F11288, F1129, F1210, F1211, F12120, F12121, F12122, F12129, F1213, F12150, F12151, F12159, F12180, F12188, F1219, F1220, F1221, F12220, F12221, F12222, F12229, F1223, F12250, F12251, F12259, F12280, F12288, F1229, F1310, F1311, F13120, F13121, F13129, F13130, F13131, F13132, F13139, F1314, F13150, F13151, F13159, F13180, F13181, F13182, F13188, F1319, F1320, F1321, F13220, F13221, F13229, F13230, F13231, F13232, F13239, F1324, F13250, F13251, F13259, F1326, F1327, F13280, F13281, F13282, F13288, F1329, F1410, F1411, F14120, F14121, F14122, F14129, F1413, F1414, F14150, F14151, F14159, F14180, F14181, F14182, F14188, F1419, F1420, F1421, F14220, F14221, F14222, F14229, F1423, F1424, F14250, F14251, F14259, F14280, F14281, F14282, F14288, F1429, F1510, F1511, F15120, F15121, F15122, F15129, F1513, F1514, F15150, F15151, F15159, F15180, F15181, F15182, F15188, F1519, F1520, F1521, F15220, F15221, F15222, F15229, F1523, F1524, F15250, F15251, F15259, F15280, F15281, F15282, F15288, F1529, F1610, F1611, F16120, F16121, F16122, F16129, F1614, F16150, F16151, F16159, F16180, F16183, F16188, F1619, F1620, F1621, F16220, F16221, F16229, F1624, F16250, F16251, F16259, F16280, F16283, F16288, F1629, F1810, F1811, F18120, F18121, F18129, F1814, F18150, F18151, F18159, F1817, F18180, F18188, F1819, F1820, F1821, F18220, F18221, F18229, F1824, F18250, F18251, F18259, F1827, F18280, F18288, F1829, F1910, F1911, F19120, F19121, F19122, F19129, F19130, F19131, F19132, F19139, F1914, F19150, F19151, F19159, F1916, F1917, F19180, F19181, F19182, F19188, F1919, F1920, F1921, F19220, F19221, F19222, F19229, F19230, F19231, F19232, F19239, F1924, F19250, F19251, F19259, F1926, F1927, F19280, F19281, F19282, F19288, F1929, O99320, O99321, O99322, O99323, O99324, O99325 |
| Epilepsy | 345 | G40 |
| Hyperlipidemia | 272 | E78 |
| Hypertension | 4011, 4019, 64200-64204, 4101, 4372 | H35031, H35032, H35033, H35039, I110, I119, I120, I129, I130, I1310, I1311, I132, I150, I151, I152, I158, I159, I161, I674, O10111, O10112, O10113, O10119, O1012, O1013, O10211, O10212, O10213, O10219, O1022, O1023, O10311, O10312, O10313, O10319, O1032, O1033, O10411, O10412, O10413, O10419, O1042, O1043, O10911, O10912, O10913, O10919, O1092, O1093, O111, O112, O113, O114, O115, O119, O161, O162, O163, O164, O165, O169, I10, I160, I169, O10011, O10012, O10013, O10019, O1002, O1003 |
| Hypotension | 458 | I95 |
| Ischemic heart disease | 412, 4110, 4111, 4130, 4131, 4139, 4142, 4143, 4144, 4148, 4149, 41000, 41001, 41002, 41010, 41011, 41012, 41020, 41021, 41022, 41030, 41031, 41032, 41040, 41041, 41042, 41050, 41051, 41052, 41060, 41061, 41062, 41070, 41071, 41072, 41080, 41081, 41082, 41090, 41091, 41092, 41181, 41189, 41400, 41401, 41402, 41403, 41404, 41405, 41406, 41407, 41412 | I200, I201, I208, I209, I2109, I2111, I2119, I2129, I213, I214, I219, I21A1, I21A9, I240, I241, I248, I2510, I252, I2542, I255, I25810, I25811, I25812, I2582, I2583, I2584, I2589, I259 |
| Osteoporosis | 733 | M80, M81 |
| Parkinson’s disease/parkinsonism | 332 | G20, G21 |
| Schizophrenia | 295 | F200, F201, F202, F205, F2081, F2089, F209, F259 |
| Stroke or cerebrovascular accident | 433, 434 | I60, I61, I62, I63, I64, G463, G464 |
| Syncope | 7802 | G9001, R054, R55, T671 |
| Urinary incontinence | 7883 | R32, R3981, N393, N394 |
| Vertigo/dizziness | 3860, 3861, 3869, 7804 | A881, H81, H82, R42, T7523 |
| Vision disorders | 360-379 | H00-H59 |

**Table S6. Detailed patient characteristics of eligible Medicare beneficiaries: Overall and by trajectory**

| **Trajectory groups*** | **All** | **A** | **B** | **C** | **D** | **E** | **F** | **G** | **H** | **I** | **J** | **K** | **L** | **ASMD^‡^** | |
| --- | --- | --- | --- | --- | --- | --- | --- | --- | --- | --- | --- | --- | --- | --- | --- |
|  |  |  |  |  |  |  |  |  |  |  |  |  |  | **Before IPTW** | **After IPTW** |
| Total | 102750 (100.0) | 17820 (17.3) | 31824 (31.0) | 24194 (23.5) | 5586 (5.4) | 4161 (4.0) | 717 (0.7) | 7004 (6.8) | 1291 (1.3) | 6351 (6.2) | 1308 (1.3) | 1771 (1.7) | 723 (0.7) |  |  |
| **Patient-level** |  |  |  |  |  |  |  |  |  |  |  |  |  |  |  |
| Age, mean (SD) | 75.5 (7.5) | 75.8 (7.5) | 76.8 (8.0) | 74.7 (7.1) | 73.4 (6.5) | 75.3 (7.2) | 73.4 (6.5) | 76.2 (7.8) | 74.0 (6.9) | 74.6 (7.0) | 73.2 (6.5) | 73.3 (6.4) | 72.2 (6.0) | 0.20 | 0.07 |
| Male, % | 33.0 | 31.6 | 32.4 | 37.0 | 36.6 | 26.7 | 31.7 | 28.7 | 33.3 | 31.8 | 33.2 | 28.9 | 28.6 | 0.07 | 0.11 |
| Race, % |  |  |  |  |  |  |  |  |  |  |  |  |  |  |  |
| White | 81.2 | 78.0 | 79.2 | 85.0 | 86.6 | 82.2 | 80.8 | 79.2 | 74.5 | 84.3 | 75.2 | 86.7 | 79.1 | 0.11 | 0.08 |
| Black | 6.1 | 8.4 | 7.6 | 4.9 | 4.4 | 4.3 | 4.5 | 4.8 | 4.7 | 3.1 | 2.8 | 2.1 | 2.9 | 0.09 | 0.05 |
| Asian | 1.9 | 2.7 | 2.5 | 1.4 | 1.1 | 1.8 | 0.7 | 1.9 | 0.7 | 1.2 | 0.8 | 0.7 | 0.6 | 0.06 | 0.09 |
| Hispanic | 8.7 | 8.8 | 8.7 | 6.7 | 5.7 | 9.5 | 12.8 | 11.9 | 18.6 | 9.5 | 19.4 | 8.4 | 16.0 | 0.13 | 0.07 |
| Others | 2.0 | 2.0 | 2.0 | 2.1 | 2.3 | 2.2 | 1.3 | 2.2 | 1.5 | 1.8 | 1.8 | 2.0 | 1.4 | 0.02 | 0.03 |
| Disability, % | 2.6 | 3.7 | 2.7 | 2.5 | 2.2 | 1.9 | 2.9 | 1.6 | 2.6 | 1.8 | 2.4 | 1.2 | 1.1 | 0.06 | 0.08 |
| Dual eligibility, % | 26.4 | 26.4 | 29.5 | 22.1 | 21.7 | 22.1 | 32.1 | 29.2 | 40.7 | 24.2 | 38.1 | 24.8 | 37.3 | 0.17 | 0.11 |
| Low-income subsidy, % | 26.3 | 26.4 | 29.3 | 21.8 | 21.6 | 23.0 | 32.8 | 29.2 | 41.6 | 24.2 | 39.2 | 24.4 | 38.0 | 0.17 | 0.11 |
| Metropolitan county, % | 83.5 | 81.0 | 83.3 | 82.7 | 86.5 | 83.6 | 81.9 | 85.7 | 86.3 | 86.1 | 89.1 | 89.9 | 89.5 | 0.10 | 0.10 |
| Resided region, % |  |  |  |  |  |  |  |  |  |  |  |  |  |  |  |
| Northeast | 12.0 | 14.2 | 13.9 | 10.8 | 8.1 | 13.0 | 7.9 | 11.4 | 8.4 | 9.4 | 7.3 | 6.8 | 6.4 | 0.10 | 0.06 |
| Midwest | 15.2 | 17.5 | 16.5 | 16.7 | 11.4 | 13.4 | 8.8 | 12.3 | 10.7 | 11.6 | 7.1 | 6.0 | 4.1 | 0.15 | 0.15 |
| South | 60.6 | 53.0 | 56.2 | 60.7 | 71.5 | 62.1 | 73.5 | 65.5 | 74.4 | 69.9 | 80.1 | 82.1 | 86.3 | 0.25 | 0.18 |
| West | 12.2 | 15.4 | 13.4 | 11.8 | 9.0 | 11.5 | 9.8 | 10.8 | 6.5 | 9.1 | 5.4 | 5.0 | 3.2 | 0.14 | 0.08 |
| Frailty, % | 10.6 | 9.3 | 13.0 | 9.1 | 7.2 | 9.4 | 10.0 | 13.7 | 13.3 | 10.0 | 9.9 | 5.9 | 5.5 | 0.11 | 0.09 |
| Comorbidities, % |  |  |  |  |  |  |  |  |  |  |  |  |  |  |  |
| Anxiety disorder | 32.5 | 27.0 | 25.6 | 25.0 | 25.6 | 57.8 | 70.9 | 55.5 | 64.3 | 53.6 | 57.6 | 47.4 | 50.9 | 0.43 | 0.13 |
| Bipolar disorder | 2.7 | 2.0 | 2.4 | 2.1 | 3.3 | 3.1 | 6.7 | 3.8 | 8.1 | 2.9 | 7.0 | 5.4 | 9.4 | 0.12 | 0.08 |
| Chronic pain | 11.9 | 12.9 | 11.8 | 10.4 | 8.7 | 14.7 | 26.6 | 14.0 | 20.1 | 10.4 | 14.3 | 10.0 | 10.4 | 0.12 | 0.05 |
| Dementia | 13.6 | 10.0 | 18.2 | 13.2 | 10.7 | 6.9 | 4.5 | 14.9 | 12.8 | 12.0 | 12.1 | 8.8 | 6.8 | 0.13 | 0.05 |
| Epilepsy | 1.9 | 1.8 | 2.2 | 1.8 | 1.3 | 1.6 | 2.1 | 2.2 | 2.6 | 1.7 | 2.1 | 1.6 | 1.0 | 0.03 | 0.05 |
| Hyperlipidemia | 72.0 | 73.1 | 72.9 | 73.6 | 65.0 | 73.6 | 72.8 | 73.3 | 69.2 | 70.4 | 62.0 | 59.1 | 50.9 | 0.14 | 0.07 |
| Hypotension | 4.8 | 5.3 | 5.3 | 4.3 | 3.7 | 4.7 | 4.6 | 5.2 | 5.2 | 4.7 | 3.1 | 2.9 | 2.5 | 0.05 | 0.05 |
| Ischemic heart disease | 27.8 | 28.3 | 29.4 | 26.7 | 22.3 | 30.2 | 30.8 | 30.3 | 28.0 | 27.4 | 23.2 | 19.8 | 16.3 | 0.09 | 0.08 |
| Osteoarthritis | 34.3 | 36.8 | 36.1 | 32.2 | 26.8 | 38.2 | 43.2 | 36.0 | 36.2 | 32.2 | 32.1 | 23.3 | 22.5 | 0.13 | 0.10 |
| Osteoporosis | 13.7 | 14.9 | 14.8 | 11.9 | 9.5 | 17.3 | 15.6 | 15.8 | 14.6 | 13.0 | 10.7 | 8.0 | 9.5 | 0.09 | 0.05 |
| Parkinson’s disease | 2.4 | 1.9 | 2.7 | 2.3 | 1.8 | 1.7 | 1.7 | 3.0 | 2.9 | 2.2 | 2.1 | 2.2 | 2.1 | 0.04 | 0.08 |
| Schizophrenia | 0.9 | 0.8 | 1.1 | 0.6 | 0.7 | 0.6 | 0.3 | 1.1 | 3.6 | 0.8 | 1.6 | 0.7 | 0.6 | 0.05 | 0.04 |
| Stroke | 6.7 | 7.0 | 7.5 | 6.9 | 5.3 | 5.4 | 5.2 | 6.5 | 5.2 | 5.6 | 3.8 | 4.1 | 2.8 | 0.07 | 0.06 |
| Syncope | 5.8 | 6.3 | 6.2 | 5.1 | 4.2 | 6.5 | 6.7 | 6.7 | 7.7 | 5.8 | 6.1 | 3.0 | 3.0 | 0.06 | 0.05 |
| Urinary incontinence | 10.5 | 11.0 | 11.4 | 10.1 | 8.9 | 10.3 | 9.6 | 11.0 | 11.6 | 9.2 | 7.5 | 7.8 | 7.6 | 0.05 | 0.07 |
| Vertigo | 14.7 | 17.6 | 15.5 | 12.6 | 9.4 | 18.6 | 16.3 | 16.3 | 16.3 | 14.0 | 10.9 | 8.4 | 8.3 | 0.11 | 0.11 |
| Vision disorder | 52.0 | 56.2 | 55.3 | 50.5 | 38.0 | 56.0 | 49.1 | 54.5 | 46.0 | 48.5 | 39.2 | 31.6 | 25.0 | 0.19 | 0.15 |
| Elixhauser Comorbidity Index, mean (SD) | 5.0 (3.0) | 5.1 (3.0) | 5.2 (3.0) | 4.8 (2.9) | 4.2 (2.8) | 5.2 (3.0) | 5.3 (3.0) | 5.3 (3.1) | 5.3 (3.1) | 4.8 (3.0) | 4.6 (3.1) | 3.8 (2.8) | 3.6 (2.7) | 0.17 | 0.07 |
| Comedications, % |  |  |  |  |  |  |  |  |  |  |  |  |  |  |  |
| ACEIs/ARBs | 42.7 | 42.2 | 44.3 | 44.3 | 36.8 | 42.6 | 41.6 | 43.0 | 41.4 | 40.6 | 37.8 | 32.5 | 30.4 | 0.08 | 0.12 |
| Antiarrhythmic agents (class 1 & 3) | 2.0 | 2.2 | 2.0 | 1.9 | 1.2 | 2.9 | 2.6 | 2.5 | 2.3 | 2.1 | 1.7 | 1.9 | 2.4 | 0.03 | 0.10 |
| Anticonvulsants | 13.8 | 11.8 | 14.2 | 12.9 | 13.2 | 13.4 | 17.2 | 16.2 | 21.4 | 14.0 | 20.6 | 15.6 | 17.8 | 0.09 | 0.08 |
| Antidementia agents | 7.9 | 4.8 | 10.2 | 8.5 | 7.1 | 3.3 | 3.1 | 8.6 | 7.8 | 7.3 | 8.4 | 6.9 | 9.3 | 0.08 | 0.05 |
| Antidiabetic agents | 18.5 | 18.1 | 19.7 | 19.7 | 18.5 | 15.0 | 15.2 | 16.5 | 17.3 | 15.7 | 16.9 | 13.5 | 14.7 | 0.06 | 0.09 |
| Antihistamines | 1.9 | 2.0 | 1.8 | 1.5 | 1.5 | 2.0 | 3.2 | 2.3 | 3.6 | 2.7 | 3.1 | 1.9 | 3.0 | 0.05 | 0.08 |
| Antiparkinsonian agents | 3.6 | 2.7 | 3.9 | 3.5 | 3.3 | 2.9 | 2.9 | 4.4 | 6.1 | 3.9 | 4.7 | 4.2 | 5.0 | 0.05 | 0.07 |
| Antipsychotics | 5.2 | 3.1 | 4.8 | 4.1 | 6.7 | 4.2 | 7.1 | 7.1 | 16.7 | 7.2 | 15.1 | 9.9 | 16.9 | 0.17 | 0.09 |
| Antithrombotic agents | 16.5 | 15.8 | 17.8 | 16.5 | 13.5 | 15.9 | 16.6 | 17.7 | 15.3 | 16.0 | 12.2 | 12.8 | 10.8 | 0.07 | 0.07 |
| Anxiolytics† | 0.8 | 0.8 | 0.8 | 0.6 | 0.9 | 0.9 | 0.6 | 0.9 | 1.5 | 0.8 | 1.4 | 1.0 | 1.4 | 0.03 | 0.05 |
| Beta blockers | 33.3 | 32.7 | 34.8 | 32.7 | 27.9 | 35.8 | 38.9 | 36.0 | 33.8 | 32.5 | 30.2 | 24.3 | 26.3 | 0.09 | 0.11 |
| Bisphosphonates | 0.5 | 0.5 | 0.5 | 0.4 | 0.5 | 0.4 | 0.3 | 0.4 | 0.3 | 0.5 | 0.5 | 0.6 | 0.3 | 0.02 | 0.04 |
| Calcium channel blockers | 23.5 | 23.5 | 25.3 | 22.8 | 19.3 | 23.5 | 25.5 | 25.0 | 21.5 | 22.6 | 21.8 | 18.8 | 15.5 | 0.07 | 0.12 |
| Diuretics | 22.5 | 21.8 | 24.2 | 22.5 | 19.2 | 21.4 | 21.2 | 23.0 | 21.7 | 20.9 | 22.4 | 17.2 | 14.1 | 0.05 | 0.08 |
| Hypnotics/ sedatives† | 4.7 | 4.2 | 4.2 | 4.1 | 4.7 | 6.1 | 7.8 | 6.0 | 7.4 | 6.3 | 8.6 | 7.5 | 8.3 | 0.08 | 0.04 |
| Lipid-modifying agents | 47.4 | 43.1 | 49.0 | 51.0 | 45.7 | 42.7 | 39.1 | 47.3 | 42.2 | 47.6 | 42.5 | 42.2 | 36.9 | 0.09 | 0.16 |
| Mood stabilizers | 1.6 | 1.0 | 1.7 | 1.4 | 1.6 | 1.4 | 1.7 | 2.6 | 3.6 | 1.7 | 3.5 | 2.4 | 2.2 | 0.06 | 0.04 |
| Muscle relaxants | 3.0 | 2.6 | 2.7 | 2.8 | 3.0 | 3.3 | 5.7 | 3.5 | 5.7 | 3.5 | 5.3 | 4.6 | 6.2 | 0.07 | 0.10 |
| NSAIDs | 8.9 | 8.4 | 8.7 | 9.2 | 8.1 | 8.1 | 10.6 | 9.2 | 13.4 | 9.5 | 13.2 | 8.4 | 11.9 | 0.05 | 0.12 |
| Opioids | 13.6 | 13.1 | 12.3 | 11.5 | 11.5 | 17.2 | 33.8 | 16.2 | 31.4 | 14.6 | 27.3 | 16.7 | 27.1 | 0.16 | 0.09 |
| Other antihypertensives | 4.7 | 4.7 | 4.8 | 4.3 | 4.0 | 5.2 | 6.7 | 5.4 | 5.7 | 4.9 | 5.3 | 3.5 | 3.3 | 0.04 | 0.06 |
| Parasympathomimetics | 0.3 | 0.2 | 0.2 | 0.3 | 0.3 | 0.3 | 0.6 | 0.3 | 0.5 | 0.3 | 0.3 | 0.2 | 0.1 | 0.02 | 0.01 |
| Psychostimulants | 0.7 | 0.4 | 0.5 | 0.6 | 1.5 | 0.8 | 2.2 | 0.8 | 1.5 | 0.8 | 1.9 | 1.5 | 3.7 | 0.07 | 0.02 |
| Systemic steroids | 4.8 | 5.4 | 4.6 | 4.2 | 3.7 | 6.2 | 5.7 | 5.5 | 6.4 | 5.1 | 5.3 | 5.3 | 3.2 | 0.04 | 0.11 |
| Vasodilators | 3.7 | 3.7 | 4.0 | 3.4 | 3.2 | 3.6 | 4.9 | 4.4 | 4.3 | 3.8 | 3.7 | 2.3 | 4.0 | 0.03 | 0.06 |
| Anticholinergic burden index, mean (SD) | 1.5 (1.9) | 1.2 (1.7) | 1.3 (1.8) | 1.2 (1.7) | 1.2 (1.6) | 2.0 (1.8) | 2.8 (2.2) | 2.2 (2.1) | 2.9 (2.4) | 2.0 (1.9) | 2.8 (2.3) | 1.8 (1.8) | 2.2 (1.9) | 0.41 | 0.17 |
| Polypharmacy, % | 10.8 | 8.9 | 11.8 | 10.4 | 8.9 | 9.8 | 13.8 | 12.7 | 15.5 | 11.2 | 14.8 | 9.5 | 11.2 | 0.08 | 0.10 |
| Healthcare utilization, mean (SD) |  |  |  |  |  |  |  |  |  |  |  |  |  |  |  |
| ED visits | 0.6 (1.3) | 0.7 (1.5) | 0.6 (1.2) | 0.5 (1.1) | 0.4 (0.9) | 0.8 (1.5) | 0.8 (1.5) | 0.8 (1.4) | 0.8 (2.3) | 0.6 (1.2) | 0.5 (1.2) | 0.3 (0.9) | 0.3 (1.0) | 0.16 | 0.08 |
| Outpatient visits | 4.9 (7.2) | 5.6 (7.7) | 5.3 (7.4) | 4.6 (6.8) | 3.2 (6.1) | 5.6 (7.8) | 5.3 (7.4) | 5.2 (7.5) | 4.8 (7.6) | 4.3 (6.8) | 3.3 (5.6) | 2.3 (5.2) | 1.7 (3.7) | 0.19 | 0.09 |
| Inpatient visits | 0.3 (0.8) | 0.3 (0.8) | 0.3 (0.8) | 0.3 (0.7) | 0.2 (0.6) | 0.3 (0.8) | 0.3 (0.7) | 0.4 (0.8) | 0.4 (0.9) | 0.3 (0.8) | 0.3 (0.7) | 0.2 (0.7) | 0.2 (0.7) | 0.07 | 0.08 |
| **Provider-level**^§^ |  |  |  |  |  |  |  |  |  |  |  |  |  |  |  |
| Male, % | 65.5 | 65.0 | 62.9 | 64.8 | 64.9 | 68.5 | 77.0 | 68.5 | 75.2 | 69.9 | 76.4 | 69.5 | 76.5 | 0.12 | 0.25 |
| Monthly no. patients receiving ADs, mean (SD) | 1.0 (1.7) | 0.6 (1.2) | 0.9 (1.6) | 1.0 (1.6) | 1.4 (2.0) | 0.9 (1.4) | 1.2 (1.7) | 1.2 (2.0) | 1.9 (3.1) | 1.3 (2.0) | 2.2 (3.4) | 1.8 (2.2) | 2.7 (3.4) | 0.27 | 0.08 |
| Monthly no. patients receiving BZDs, mean (SD) | 0.7 (1.3) | 0.4 (0.8) | 0.5 (1.1) | 0.6 (1.1) | 0.9 (1.4) | 0.7 (1.2) | 1.1 (1.6) | 1.0 (1.8) | 1.7 (2.9) | 1.0 (1.6) | 1.9 (3.3) | 1.4 (1.7) | 2.3 (3.1) | 0.32 | 0.11 |
| Monthly no. AD fills, mean (SD) | 10.5 (23.8) | 6.3 (14.0) | 8.8 (20.0) | 9.8 (20.6) | 15.6 (28.2) | 9.5 (18.9) | 13.8 (22.9) | 13.1 (28.2) | 24.2 (50.5) | 14.3 (28.1) | 29.9 (61.3) | 21.3 (30.4) | 36.3 (59.7) | 0.27 | 0.06 |
| Monthly no. BZD fills, mean (SD) | 6.1 (19.0) | 3.2 (9.4) | 4.4 (13.1) | 4.9 (12.0) | 7.8 (17.6) | 6.7 (17.6) | 11.4 (20.0) | 9.2 (26.2) | 21.7 (56.4) | 9.5 (23.6) | 26.3 (66.6) | 13.5 (21.8) | 29.3 (58.7) | 0.29 | 0.06 |
| Monthly AD dose, mean (SD) | 10.2 (22.9) | 5.9 (13.3) | 8.1 (18.2) | 9.4 (19.4) | 16.3 (30.1) | 9.4 (19.4) | 14.3 (24.8) | 12.4 (26.0) | 24.4 (51.2) | 14.1 (27.8) | 29.8 (58.5) | 22.5 (32.1) | 38.8 (57.7) | 0.29 | 0.07 |
| Monthly BZD dose, mean (SD) | 6.8 (42.6) | 3.2 (18.0) | 4.2 (24.5) | 4.8 (21.1) | 8.0 (31.8) | 7.4 (41.8) | 15.5 (39.9) | 10.0 (51.0) | 37.9 (162.8) | 10.8 (55.5) | 45.3 (180.6) | 14.6 (28.2) | 45.4 (147.8) | 0.20 | 0.04 |
| Specialty, % |  |  |  |  |  |  |  |  |  |  |  |  |  |  |  |
| General internal medicine | 66.1 | 63.9 | 66.9 | 69.0 | 64.5 | 69.1 | 60.4 | 66.3 | 58.2 | 66.7 | 53.4 | 58.3 | 41.2 | 0.13 | 0.10 |
| Psychiatry | 1.0 | 0.7 | 0.7 | 0.8 | 1.6 | 0.7 | 1.8 | 1.1 | 2.4 | 1.2 | 3.1 | 2.6 | 5.8 | 0.09 | 0.05 |
| Others | 32.9 | 35.4 | 32.4 | 30.2 | 34.0 | 30.1 | 37.8 | 32.6 | 39.4 | 32.0 | 43.6 | 39.1 | 53.0 | 0.11 | 0.12 |

**Abbreviations:** ACEI: angiotensin converting enzyme inhibitor, AD: antidepressants, ARB: angiotensin receptor blocker, ASMD: absolute standardized mean difference, BZD: benzodiazepine, ED: emergency department, No.: number of, NSAID: nonsteroidal anti-inflammatory drug, SD: standard deviation

*Trajectory groups: A: low discontinuing AD (17.3% of the cohort); B: low declining AD (31.0%); C: moderate increasing AD (23.5%); D: high increasing AD (5.4%); E: low discontinuing AD/very-low declining BZD (4.0%); F: low discontinuing AD/low declining BZD (0.7%); G: low declining AD/very-low declining BZD (6.8%); H: low declining AD/low declining BZD (1.3%); I: moderate increasing AD/very-low declining BZD (6.2%); J: moderate increasing AD/low stable BZD (1.3%); K: very-high increasing AD/very-low stable BZD (1.7%); L: very-high increasing AD/low-dose increasing BZD (0.7%).

To facilitate the labeling of AD and BZD dose levels for each trajectory, we defined AD use as: negligible (standardized daily dose [SDD] < 0.1 defined daily dose [DDD]), very low (0.1 to < 0.5 DDD), low (0.5 to <1 DDD), moderate (1 to <1.5 DDD), high (1.5 to <2 DDD) and very high dose (≥ 2 DDD). Similarly, we defined BZD use as negligible (SDD < 1 diazepam milligram equivalent [DME]), very-low (< 5 DME), low (5 to <10 DME), moderate (10 to <15 DME), high (15 to <20 DME) and very-high dose (≥20 DME). We defined a “discontinuing” pattern as a dose reduction to the negligible level, “declining” or “increasing” patterns when the absolute change exceeded 0.1 DDD for ADs or 1 DME for BZDs, and “stable” when changes remained below these thresholds.
^†^Benzodiazepines were not included in the medication classes of anxiolytics and hypnotics/sedatives.

^‡^Median ASMD of 66 ASMDs from group comparisons (the number of 2-combinations from 12 trajectories: $C_{2}^{12}=66;($e.g., group A vs B, A vs C).

^§^The provider was identified as the first physician who recorded the diagnosis of depression in the patient’s medical records. The missingness of the provider-related factors was 22.1%.

**Table S7. Trajectories of Antidepressant and Benzodiazepine Utilization Patterns and Risk of Falls and Related Injuries: Subgroup and Sensitivity analyses**

| **Trajectory group*** | **Adjusted Hazard Ratio (95% Confidence Interval)** | | | | | | |
| --- | --- | --- | --- | --- | --- | --- | --- |
|  | **Main analysis (n=102,750)** | **Patients with frailty (n=10,885)** | **Patients without frailty (n=91,865)** | **Patients with dementia (n=14,016)** | **Patients without dementia (n=88,734)** | **Follow-up shortened to 183 days (n=102,750)** | **FRI identified using the inclusive algorithm (n=102,750)** |
| A | Reference | Reference | Reference | Reference | Reference | Reference | Reference |
| B | 1.11 (1.04, 1.19) | 1.16 (0.98, 1.37) | 1.11 (1.03, 1.19) | 1.13 (0.98, 1.31) | 1.11 (1.03, 1.20) | 1.12 (1.03, 1.23) | 1.08 (1.04, 1.13) |
| C | 1.24 (1.16, 1.32) | 1.39 (1.17, 1.65) | 1.22 (1.13, 1.31) | 1.34 (1.15, 1.55) | 1.21 (1.12, 1.31) | 1.27 (1.16, 1.39) | 1.18 (1.13, 1.23) |
| D | 1.29 (1.16, 1.42) | 1.25 (0.98, 1.61) | 1.30 (1.16, 1.45) | 1.23 (0.99, 1.53) | 1.31 (1.17, 1.46) | 1.31 (1.15, 1.49) | 1.25 (1.17, 1.33) |
| E | 0.84 (0.73, 0.96) | 0.91 (0.66, 1.27) | 0.81 (0.70, 0.95) | 1.09 (0.82, 1.44) | 0.77 (0.66, 0.91) | 0.80 (0.67, 0.97) | 0.99 (0.91, 1.08) |
| F | 1.09 (0.78, 1.52) | 1.03 (0.42, 2.57) | 1.11 (0.78, 1.59) | 0.30 (0.06, 1.59) | 1.28 (0.91, 1.79) | 1.39 (0.94, 2.05) | 1.16 (0.94, 1.42) |
| G | 1.15 (1.04, 1.27) | 1.18 (0.91, 1.52) | 1.15 (1.03, 1.28) | 1.38 (1.12, 1.70) | 1.09 (0.98, 1.23) | 1.20 (1.06, 1.37) | 1.15 (1.08, 1.23) |
| H | 1.27 (1.02, 1.60) | 0.49 (0.22, 1.09) | 1.43 (1.13, 1.81) | 0.80 (0.42, 1.51) | 1.41 (1.11, 1.80) | 1.26 (0.93, 1.70) | 1.18 (1.02, 1.37) |
| I | 1.28 (1.16, 1.41) | 1.41 (1.10, 1.80) | 1.26 (1.13, 1.40) | 1.44 (1.16, 1.77) | 1.24 (1.10, 1.39) | 1.40 (1.24, 1.59) | 1.30 (1.22, 1.38) |
| J | 1.71 (1.41, 2.08) | 0.89 (0.42, 1.90) | 1.86 (1.51, 2.28) | 0.90 (0.48, 1.69) | 1.92 (1.56, 2.37) | 1.83 (1.42, 2.35) | 1.62 (1.42, 1.84) |
| K | 1.39 (1.18, 1.64) | 1.57 (1.08, 2.30) | 1.36 (1.13, 1.63) | 1.13 (0.77, 1.65) | 1.47 (1.22, 1.76) | 1.42 (1.15, 1.76) | 1.42 (1.28, 1.58) |
| L | 1.96 (1.53, 2.49) | 1.69 (0.79, 3.62) | 2.05 (1.58, 2.64) | 3.89 (2.33, 6.49) | 1.77 (1.35, 2.33) | 1.89 (1.37, 2.60) | 1.75 (1.48, 2.07) |

*Trajectory groups: A: low discontinuing AD (17.3% of the cohort); B: low declining AD (31.0%); C: moderate increasing AD (23.5%); D: high increasing AD (5.4%); E: low discontinuing AD/very-low declining BZD (4.0%); F: low discontinuing AD/low declining BZD (0.7%); G: low declining AD/very-low declining BZD (6.8%); H: low declining AD/low declining BZD (1.3%); I: moderate increasing AD/very-low declining BZD (6.2%); J: moderate increasing AD/low stable BZD (1.3%); K: very-high increasing AD/very-low stable BZD (1.7%); L: very-high increasing AD/low-dose increasing BZD (0.7%).

To facilitate the labeling of AD and BZD dose levels for each trajectory, we defined AD use as: negligible (standardized daily dose [SDD] < 0.1 defined daily dose [DDD]), very low (0.1 to < 0.5 DDD), low (0.5 to <1 DDD), moderate (1 to <1.5 DDD), high (1.5 to <2 DDD) and very high dose (≥ 2 DDD). Similarly, we defined BZD use as negligible (SDD < 1 diazepam milligram equivalent [DME]), very-low (< 5 DME), low (5 to <10 DME), moderate (10 to <15 DME), high (15 to <20 DME) and very-high dose (≥20 DME). We defined a “discontinuing” pattern as a dose reduction to the negligible level, “declining” or “increasing” patterns when the absolute change exceeded 0.1 DDD for ADs or 1 DME for BZDs, and “stable” when changes remained below these thresholds.


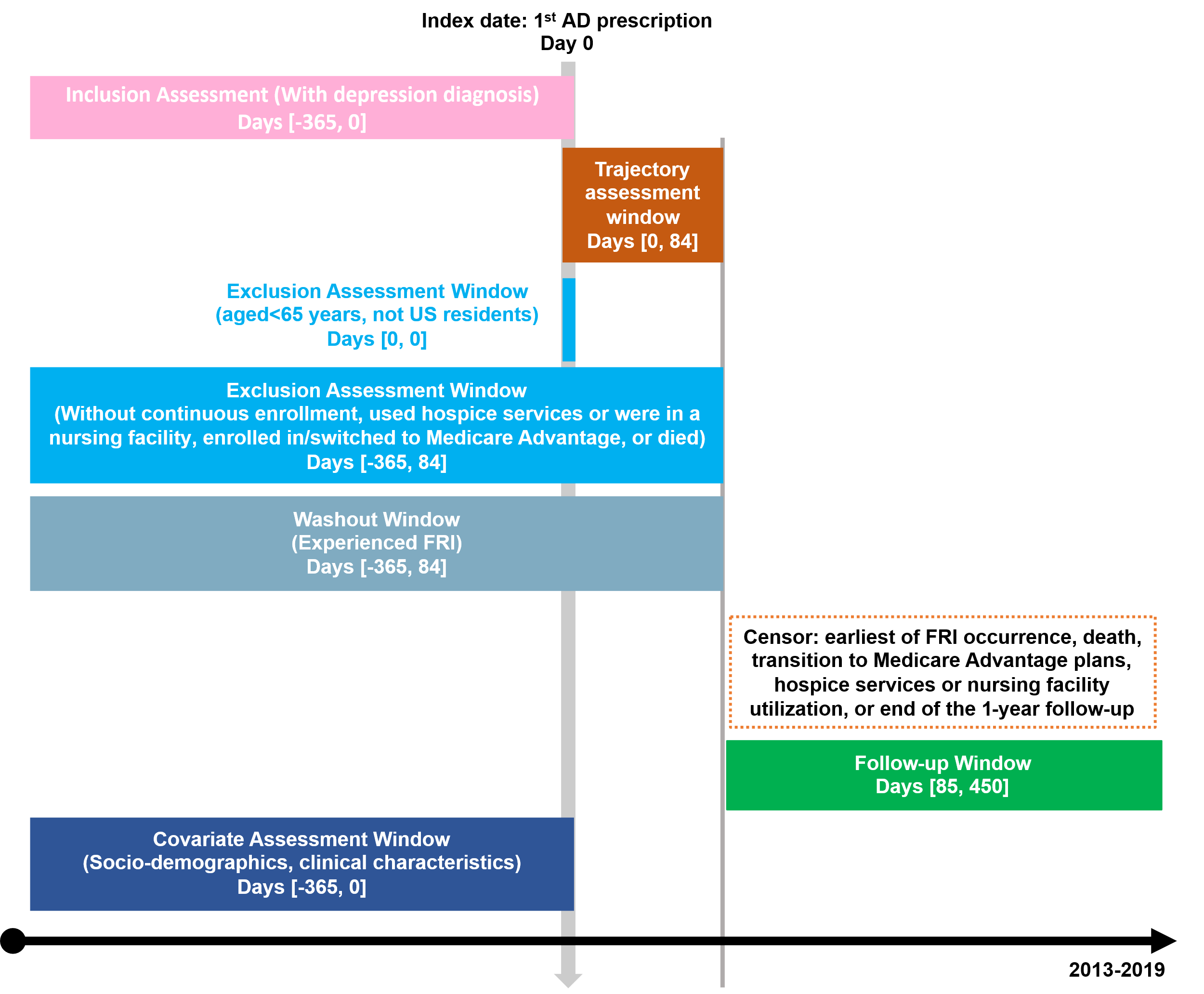


**Figure S1. Study Design Schematic Diagram**

**Abbreviations:** AD: antidepressants, FRI: falls and related injuries


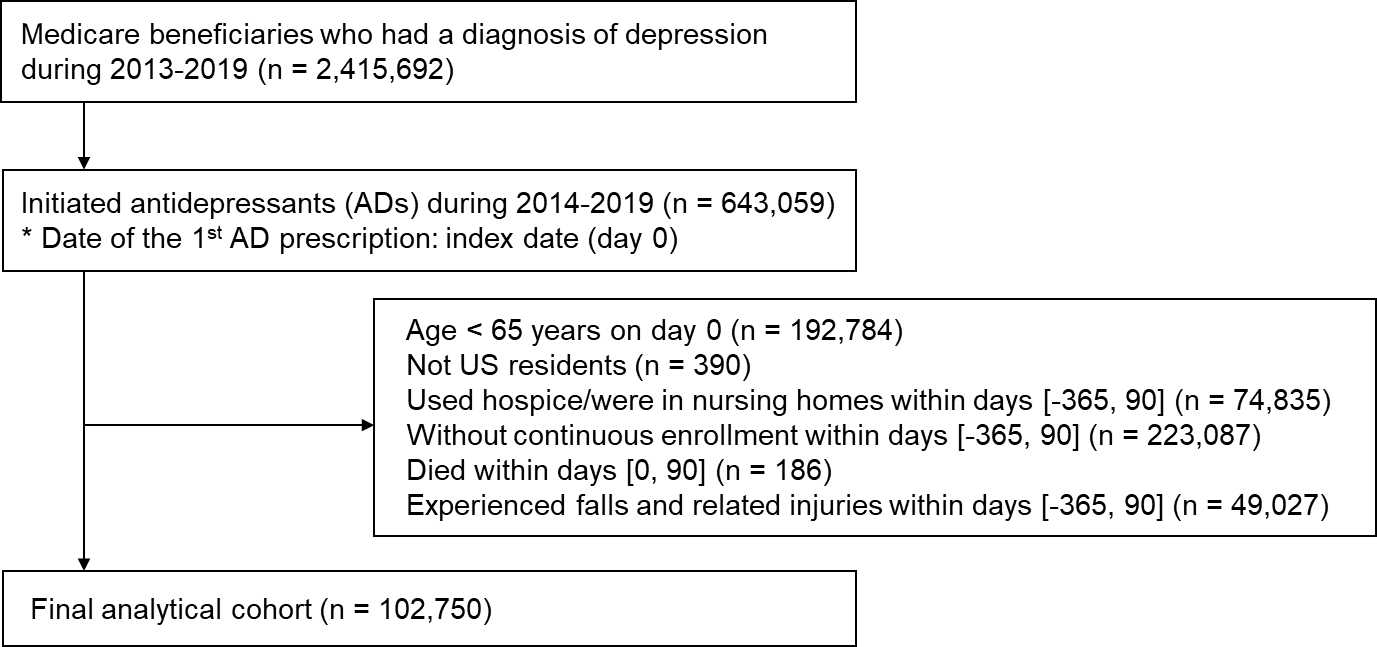


**Figure S2. Cohort Selection Flowchart**
